# Comparison of Immunogenicity and Reactogenicity of Five Primary Series of COVID-19 Vaccine Regimens against Circulating SARS-CoV-2 Variants of Concern among Healthy Thai Populations

**DOI:** 10.3390/vaccines11030564

**Published:** 2023-03-01

**Authors:** Tavitiya Sudjaritruk, Oramai Mueangmo, Jutamad Saheng, Poramed Winichakoon, Parichat Salee, Worawan Wongjak, Tanachot Chaito, Jutarat Praparattanapan, Khanuengnit Nuket, Nuttarika Solai, Jiraprapa Wipasa, Kriangkrai Chawansuntati, Romanee Chaiwarith

**Affiliations:** 1 Division of Infectious Diseases, Department of Pediatrics, Faculty of Medicine, Chiang Mai University, Chiang Mai 50200, Thailand; 2 Clinical and Molecular Epidemiology of Emerging and Re-Emerging Infectious Diseases Research Cluster, Faculty of Medicine, Chiang Mai University, Chiang Mai 50200, Thailand; 3 Division of Infectious Diseases and Tropical Medicine, Department of Internal Medicine, Faculty of Medicine, Chiang Mai University, Chiang Mai 50200, Thailand; 4 Research Institute for Health Sciences, Chiang Mai University, Chiang Mai 50200, Thailand

**Keywords:** CoronaVac, ChAdOx1 nCoV-19 vaccine, heterologous COVID-19 vaccination, homologous COVID-19 vaccination, Oxford-AstraZeneca COVID-19 vaccine, Pfizer-BioNTech COVID-19 vaccine

## Abstract

To compare immunogenicity and reactogenicity of five COVID-19 vaccine regimens against wild-type SARS-CoV-2 and variants of concern (VoCs) among Thai populations, a prospective cohort study was conducted among healthy participants aged ≥18 years who had never been infected with COVID-19 and were scheduled to get one of the five primary series of COVID-19 vaccine regimens, including CoronaVac/CoronaVac, AZD1222/AZD1222, CoronaVac/AZD1222, AZD1222/BNT162b2, and BNT162b2/BNT162b2. Anti-receptor binding domain (anti-RBD-WT) IgG and neutralizing antibody (NAb-WT) against wild-type SARS-CoV-2 were measured at pre-prime, post-prime, and post-boost visits. NAb against VoCs (NAb-Alpha, NAb-Beta, NAb-Delta, and NAb-Omicron) were assessed at the post-boost visit. Adverse events (AEs) following vaccination were recorded. A total of 901 participants (CoronaVac/CoronaVac: 332, AZD1222/AZD1222: 221, CoronaVac/AZD1222: 110, AZD1222/BNT162b2: 128, and BNT162b2/BNT162b2: 110) were enrolled. Anti-RBD-WT IgG and NAb-WT levels increased substantially after each vaccine dose. At the post-boost visit, BNT162b2/BNT162b2 induced the highest GMC of anti-RBD-WT IgG level (1698 BAU/mL), whereas AZD1222/BNT162b2 induced the highest median NAb-WT level (99% inhibition). NAb levels against VoCs, particularly the Omicron strain, were markedly attenuated for all vaccine regimens (*p* < 0.001). Overall, no serious AEs following vaccination were observed. All five primary series of COVID-19 vaccine regimens were well-tolerated and elicited robust antibody responses against wild-type SARS-CoV-2 but had attenuated responses against VoCs, particularly the Omicron strain, among healthy Thai populations.

## 1. Introduction

In the light of the global pandemic of coronavirus disease in 2019 (COVID-19), vaccination against the severe acute respiratory syndrome coronavirus 2 (SARS-CoV-2) is the most efficient and vital strategy to protect against infection and prevent severe disease, hospitalization, and death [1,2,3]. As of January 2023, 50 vaccines based on different platforms, including inactivated whole viruses, viral vectors, nucleic acids, and protein subunits, with different efficacies, have been authorized or licensed for use in at least one country, of which 11 have been granted emergency use listing by the World Health Organization (WHO) [4]. To date, a total of 13.3 billion doses of the COVID-19 vaccine have been administered worldwide, and 1.2 million are administered each day. Overall, 69% of the world’s population has received at least one dose of the COVID-19 vaccine, whereas only 26% of people in low-income countries have received at least one dose [5].

During the initial phase of the COVID-19 vaccination rollout, most of the approved vaccines followed a homologous prime-boost regimen, in which the same type of vaccine was administered for a priming and a booster dose. However, regarding the concerns of serious adverse events following immunization (AEFIs), waning of vaccine-elicited immunity, new emerging variants of concern (VoCs), and intermittent vaccine supply shortages, a heterologous prime-boost vaccine schedule, which is the use of different types of vaccines for a priming and a booster dose, has gained increasing interest as an alternative vaccine regimen, particularly for low- and middle-income countries. There is growing evidence that this vaccine regimen could provide robust immune responses with safe reactogenicity profiles [6,7,8].

Antibodies have been established as a clear correlate of protection against SARS-CoV-2 infection after vaccination [9,10]. However, the immunogenicity of each COVID-19 vaccine regimen varies widely across studies due to variation in the study population, study setting, vaccine dosage and immunization schedule, laboratory measurement of vaccine-elicited antibodies, and predominant SARS-CoV-2 variants during the study period, which caused challenges in comparing the immunogenicity across vaccine regimens. To date, few head-to-head comparative studies have been conducted to assess antibody responses to diverse vaccine regimens [11]. Additionally, the fast-track evaluation and approval of currently available COVID-19 vaccines have made it necessary to demonstrate the real-world safety and reactogenicity of the vaccines [12].

In Thailand, inactivated whole virus (e.g., CoronaVac) and adenovirus-vectored vaccines (e.g., AZD1222) were among the first vaccine platforms authorized for the general population during the early phase of the COVID-19 pandemic, followed by mRNA vaccines (e.g., BNT162b2). The recommendations for a primary series of the COVID-19 vaccine regimen have been continuously updated by the Ministry of Public Health of Thailand (MOPH, Thailand), according to the availability of vaccine supplies in the country [13]. As of 2 December 2022, approximately 83% of the Thai population have received at least one dose, and 78% have completed a two-dose primary vaccine course [14]. This study aimed to compare the immunogenicity and safety profiles of five primary series of COVID-19 vaccine regimens against wild-type SARS-CoV-2 and other important circulating VoCs among healthy Thai populations.

## 2. Materials and Methods

### 2.1. Study Design and Study Population

This is a single-center prospective cohort study carried out from June 2021 to January 2022 at the Faculty of Medicine, Chiang Mai University, Chiang Mai, Thailand. According to the WHO report, there had been 2290 to 23,418 confirmed COVID-19 cases with 9 to 301 COVID-19-related deaths per day in Thailand during the study period [15]. Healthy participants aged 18 years and older who had neither a confirmed diagnosis of COVID-19 nor previously received any vaccines against SARS-CoV-2 and were scheduled to get one of the five primary series of COVID-19 vaccine regimens, including CoronaVac/CoronaVac, AZD1222/AZD1222, CoronaVac/AZD1222, AZD1222/BNT162b2, or BNT162b2/BNT162b2, were enrolled. Participants with a history of vaccine-associated hypersensitivity, unstable or uncontrolled comorbidities, an immunocompromised state, receiving immunosuppressive agents, or pregnancy were excluded. The study was approved by the Research Ethics Committee of the Faculty of Medicine, Chiang Mai University (approval numbers: 187/2021; 232/2021; 344/2021; and 413/2021). All participants provided written informed consent prior to study enrollment.

### 2.2. Vaccination Schedule and Vaccine Administration

During the study period, the MOPH of Thailand recommended several primary series of COVID-19 vaccine regimens, including homologous prime-boost regimen of CoronaVac (SINOVAC CoronaVac^®^, 3 weeks apart), AZD1222 (ChAdOx1 nCoV-19; Vaxzevria^TM^, 12 weeks apart), and BNT162b2 (Pfizer-BioNTech COVID-19 vaccine; Comirnaty^®^, 3 weeks apart), as well as heterologous prime-boost regimen of CoronaVac/AZD1222 (3–4 weeks apart), and AZD1222/BNT162b2 (4–8 weeks apart) [13]. Each dose of vaccine was given intramuscularly. All participants were observed at the vaccination center for 30 min to monitor any immediate AEFIs.

### 2.3. Data Collection and Safety Assessment

At enrollment, participant characteristics and the history of administration of other vaccines within the past 6 months were collected. Any immediate adverse events (AEs) observed within 30 min following vaccination were recorded. Additionally, all participants were instructed to self-assess and self-report any AEs, both local and systemic, using an electronic vaccine diary for seven days after each dose of vaccine. The solicited local AEs included pain, erythema, and swelling at the injection site, whereas the solicited systemic AEs included fatigue, headache, fever, paresthesia, and dizziness.

### 2.4. Blood Collection

Blood samples were collected at the pre-prime (before the priming dose), post-prime (3–4 weeks after the priming dose, depending on the vaccine regimen), and post-boost visits (4 weeks after the booster dose). Sera were extracted, aliquoted, and stored at −70 °C until the laboratory analyses.

### 2.5. Serological Testing

#### 2.5.1. Chemiluminescent Microparticle Immunoassay for Wild-Type SARS-CoV-2 RBD IgG Antibody

All sera were analyzed for immunoglobulin G (IgG) antibodies to the receptor-binding domain of the S1 subunit of wild-type SARS-CoV-2 spike protein (anti-RBD-WT IgG) using the SARS-CoV-2 IgG II Quant assay (kit catalog number: 06S6122) on the Alinity i System (Abbott Diagnostics, Chicago, IL, USA), according to the manufacturer’s instructions. The chemiluminescent microparticle immunoassay (CMIA) technique was used in this automated immunoassay for the quantitative determination of anti-RBD-WT IgG level. In brief, the participant’s serum, SARS-CoV-2 antigen-coated paramagnetic microparticles, and assay diluent were combined and incubated. The anti-RBD-WT IgG presenting in the participant’s serum was bound to the SARS-CoV-2 antigen-coated microparticles. Following the addition of an anti-human IgG acridinium-labeled conjugate, the resulting chemiluminescent reaction was measured as a relative light unit (RLU). The amount of anti-RBD-WT IgG for each participant was determined by comparing the chemiluminescent RLU to the IgG II calibrator RLU. The unit of anti-RBD-WT IgG antibody (arbitrary unit [AU]/mL) was converted to binding antibody units (BAU)/mL using the equation suggested by the manufacturer (BAU/mL = 0.142 * AU/mL). The cut-off value of ≥50 AU/mL or ≥7.1 BAU/mL was considered seropositive.

#### 2.5.2. Surrogate Virus Neutralization Test for Wild-Type SARS-CoV-2

Neutralizing antibodies against wild-type SARS-CoV-2 (NAb-WT) were measured with the surrogate virus neutralization test (sVNT) in enzyme-linked immunosorbent assay (ELISA) format, using the SARS-CoV-2 NeutraLISA (Euroimmun AG, Lübeck, Germany). The result was shown in percentage of inhibition (% inhibition), and the cut-off value of ≥35% was defined as detectable neutralizing activity.

#### 2.5.3. In-House Surrogate Virus Neutralization Test for SARS-CoV-2 Variants of Concern

A subset of 92 and 120 serum samples from randomly selected participants who received CoronaVac/CoronaVac and AZD1222/AZD1222, together with all serum samples from participants receiving CoronaVac/AZD1222 (106 samples), AZD1222/BNT162b2 (107 samples), and BNT162b2/BNT162b2 (94 samples) which were collected at the post-boost visit were analyzed for NAb-WT and NAb against VoCs, including NAb-Alpha (B.1.1.7), NAb-Beta (B.1.351), NAb-Delta (B.1.617.2), and NAb-Omicron (B.1.1.529, except participants receiving CoronaVac/CoronaVac), using an in-house sVNT which was modified from the method previously described by Tan CW, et al. [16]. In brief, sera diluted 1:4 were incubated with horseradish peroxidase-conjugated receptor binding domain protein (Genscript, Piscataway, USA) for 30 min before being added to 96-well Maxisorp ELISA immunoplates (Thermo scientific, Roskilde, Denmark) pre-coated with 200 ng angiotensin-converting enzyme-2 (GenScript, Piscataway, NJ, USA). Plates were incubated at 37 °C for 1 h. After washing with 0.05% Tween-20 (Calbiochem, Gibbstown, NJ, USA) in phosphate buffer saline, 50 µL of tetramethylbenzidine substrate solution (Life Technologies, Frederick, MD, USA) was added. The enzymatic reaction was allowed to proceed for 30 min before 50 µL of 0.2 M sulfuric acid was added to stop the reaction. The absorbance was read at 450 nm on a CLARIOstar^®^ microtiter plate reader (Ortenberg, Germany). The result was reported as percentage of inhibition (% inhibition), and the cut-off value of ≥30% was considered detectable neutralizing activity against each SARS-CoV-2 strain.

### 2.6. Statistical Analysis

Participant characteristics and AEFIs within seven days after each dose of vaccine were presented as number (percentage) and median (interquartile range [IQR]) for categorical and continuous variables, respectively. The comparisons of such parameters across five COVID-19 vaccine regimens were performed using the Chi-squared test or Fisher’s exact test (if less than five observations) for categorical variables, and Wilcoxon rank-sum test for continuous variables.

Anti-RBD-WT IgG level was presented as geometric mean concentration (GMC) and 95% confidence interval (95% CI), and NAb titer against each SARS-CoV-2 strain (NAb-WT, NAb-Alpha, NAb-Beta, NAb-Delta, and NAb-Omicron) was reported as median and IQR. The comparisons of GMCs of anti-RBD-WT IgG between study visits within each vaccine regimen were performed using the mixed-effects log-linear regression model, whereas the comparisons between indicated vaccine regimens were conducted using the log-linear regression model. The magnitude of the difference was summarized with a geometric mean ratio (GMR) and 95% CI. In addition, the comparisons of NAb between study visits and between SARS-CoV-2 strains within each vaccine regimen were conducted using the Wilcoxon signed-rank test, while the comparisons between the indicated vaccine regimens were performed using the median regression analysis. The magnitudes of the differences were summarized with a median difference and an IQR. For the proportion of seropositive participants, the comparisons between study visits and between SARS-CoV-2 strains within each vaccine regimen were conducted using the McNemar’s test, whereas the comparisons between indicated vaccine regimens were performed using the Chi-squared test. The magnitudes of the differences were summarized with a proportion difference and 95% CI. Furthermore, within each vaccine regimen, the log-linear regression analyses and the Wilcoxon rank-sum tests were performed to compare anti-RBD-WT IgG levels as well as NAb levels against wild-type SARS-CoV-2 and other VoCs between adult and elderly participants, respectively. All statistical analyses were conducted using Stata statistical software, version 17 (StataCorp LP, College Station, TX, USA), and GraphPad Prism, version 9.0 (GraphPad, San Diego, CA, USA). A two-sided *p* < 0.05 was considered statistically significant.

## 3. Results

### 3.1. Participant Characteristics

A total of 901 participants were enrolled, of whom 332 participants receiving CoronaVac/CoronaVac (median age: 39 years; male sex: 27%); 221 participants receiving AZD1222/AZD1222 (median age: 63 years; male sex: 37%); 110 participants receiving CoronaVac/AZD1222 (median age: 60 years; male sex: 44%); 128 participants receiving AZD1222/BNT162b2 (median age: 52 years; male sex: 34%); and 110 participants receiving BNT162b2/BNT162b2 vaccine regimens (median age: 29 years; male sex: 25%) (Table 1) (Supplementary Figure S1). Notably, 44 (20%), 27 (25%), 1 (1%) and 3 (3%) participants in AZD1222/AZD1222, CoronaVac/AZD1222, AZD1222/BNT162b2, and BNT162b2/BNT162b2 groups reported having received other licensed vaccines within the past 6 months. Participant characteristics, stratified by COVID-19 vaccine regimen, are summarized in Table 1.

### 3.2. Anti-SARS-CoV-2 RBD IgG against Wild-Type SARS-CoV-2 Following a Primary Series of COVID-19 Vaccination

At the pre-prime visit, 1–2% of participants in each vaccine group were seropositive against wild-type SARS-CoV-2. At the post-prime visit, approximately half of participants receiving CoronaVac/CoronaVac (60%) and CoronaVac/AZD1222 (53%); almost all participants receiving AZD1222/AZD1222 (96%) and AZD1222/BNT162b2 (96%); and all participants receiving BNT162b2/BNT162b2 (100%) vaccine regimens demonstrated anti-RBD-WT IgG seroconversion. For the first four vaccine regimens, the proportion of seropositive participants significantly increased to 99-100% at the post-boost visit (*p* < 0.05) (Supplementary Table S1). Notably, anti-RBD-WT IgG seroconversion patterns showed the same parallel trend for adult (aged < 60 years) and elderly participants (aged *≥* 60 years) receiving AZD1222/AZD1222, CoronaVac/AZD1222, and AZD1222/BNT162 vaccine regimens (Supplementary Table S1).

**Figure 1:**
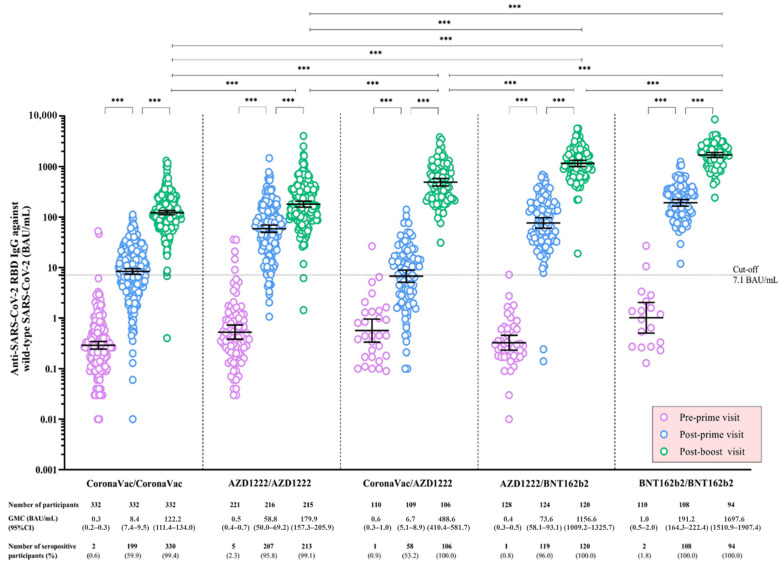
Anti-SARS-CoV-2 receptor-binding domain immunoglobulin G against wild-type SARS-CoV-2 following a primary series of COVID-19 vaccination, stratified by the COVID-19 vaccine regimen. Data points are the reciprocals of individual participants. The geometric mean concentrations (GMC) and the corresponding 95% confidence intervals (95% CI) of anti-SARS-CoV-2 receptor-binding domain immunoglobulin G against wild-type SARS-CoV-2 among all participants at the pre-prime, post-prime, and post-boost visits are shown as black solid lines. Black dotted lines indicate the cut-off values. The mixed-effects log-linear regression analysis was performed to compare the GMCs of anti-SARS-CoV-2 receptor-binding domain immunoglobulin G between visits within each COVID-19 vaccine regimen. The log-linear regression analysis was performed to compare the GMCs of anti-SARS-CoV-2 receptor-binding domain immunoglobulin G between COVID-19 vaccine regimens. *** indicates *p* < 0.001 for the comparison of the GMCs of anti-SARS-CoV-2 receptor-binding domain immunoglobulin G between indicated visits within each vaccine regimen, and between indicated vaccine regimens. Abbreviations: AZD1222 vaccine, the Oxford-AstraZeneca COVID-19 vaccine; BAU, binding antibody unit; BNT162b2, Pfizer-BioNTech vaccine; COVID-19, coronavirus disease 2019; GMC, geometric mean concentration; IgG, immunoglobulin G; RBD, receptor-binding domain; SARS-CoV-2, severe acute respiratory syndrome coronavirus-2; 95% CI, 95% confidence interval.

Regarding anti-RBD-WT IgG, the levels significantly increased after a priming and a booster dose in all vaccine regimens (*p* < 0.001). At the post-boost visit, the GMCs of anti-RBD-WT IgG were highest among participants receiving BNT162b2/BNT162b2 (1698 BAU/mL), followed by AZD1222/BNT162b2 (1157 BAU/mL), CoronaVac/AZD1222 (489 BAU/mL), AZD1222/AZD1222 (180 BAU/mL), and CoronaVac/CoronaVac (122 BAU/mL), respectively (*p* < 0.001) (Table 2) (Figure 1). The stratified analyses by participant’s age group yielded similar antibody response patterns for adults and elderly participants receiving AZD1222/AZD1222, CoronaVac/AZD1222, and AZD1222/BNT162 vaccine regimens (Table 2) (Supplementary Figure S2). Notably, at the post-boost visit, the anti-RBD-WT IgG level was significantly higher among adult participants in the AZD1222/AZD122 group (*p* < 0.001) and had a trend toward statistical significance among those in the CoronaVac/AZD1222 (*p* = 0.09) and AZD1222/BNT162b2 (*p* = 0.07) groups.

### 3.3. Neutralizing Antibody against Wild-Type SARS-CoV-2 Following a Primary Series of COVID-19 Vaccination

At the pre-prime visit, none of the participants in all five vaccine groups had a detectable NAb-WT. At the post-prime visit, 1% and 2% of participants receiving CoronaVac/CoronaVac and CoronaVac/AZD1222; 52% and 53% of those receiving AZD1222/AZD1222 and AZD1222/BNT162b2; and 81% of those receiving BNT162b2/BNT162b2 vaccine regimens developed a positive NAb-WT, respectively. The proportion of participants having a detectable NAb-WT significantly increased to 83% and 88% in CoronaVac/CoronaVac and AZD1222/AZD1222 (*p* < 0.001) and to 97%, 99%, and 100% in CoronaVac/AZD1222, AZD1222/BNT162b2, and BNT162b2/BNT162b2 vaccine groups (*p* < 0.001) at the post-boost visit, respectively (Supplementary Table S2). Of note, NAb-WT seroconversion patterns for adult and elderly participants receiving AZD1222/AZD1222, CoronaVac/AZD1222, and AZD1222/BNT162 vaccine regimens revealed similar findings (Supplementary Table S2).

**Figure 2:**
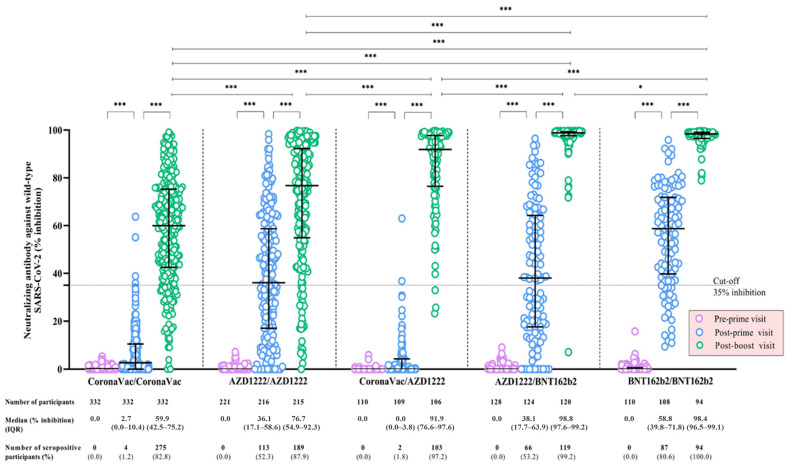
Neutralizing antibody against wild-type SARS-CoV-2 following a primary series of COVID-19 vaccination, stratified by the COVID-19 vaccine regimen. Data points are the reciprocals of individual participants. The median values of neutralizing antibodies (NAb) and the corresponding interquartile ranges (IQR) against wild-type SARS-CoV-2 among all participants at the pre-prime, post-prime, and post-boost visits are shown as black solid lines. Black dotted lines indicate the cut-off values. The Wilcoxon signed-rank test was performed to compare the medians of NAb against wild-type SARS-CoV-2 between visits within each COVID-19 vaccine regimen. The median regression analysis was performed to compare the medians of NAb against wild-type SARS-CoV-2 between COVID-19 vaccine regimens. * indicates *p* < 0.05; and *** indicates *p* < 0.001 for the comparison of NAb against wild-type SARS-CoV-2 between visits within each vaccine regimen and between indicated vaccine regimens. Abbreviations: AZD1222 vaccine, the Oxford-AstraZeneca COVID-19 vaccine; BNT162b2, Pfizer-BioNTech vaccine; COVID-19, coronavirus disease 2019; IQR, interquartile range; NAb, neutralizing antibody; SARS-CoV-2, severe acute respiratory syndrome coronavirus-2.

The NAb-WT levels of participants significantly increased after each vaccine dose for all vaccine regimens (*p* < 0.001). At the post-boost visit, the median NAb-WT levels were highest among participants receiving AZD1222/BNT162b2 (99% inhibition), followed by BNT162b2/BNT162b2 (98% inhibition), CoronaVac/AZD1222 (92% inhibition), AZD1222/AZD1222 (77% inhibition), and CoronaVac/CoronaVac (60% inhibition), respectively (*p* < 0.05) (Table 3) (Figure 2). The trajectory patterns of NAb-WT responses for adult and elderly participants receiving AZD1222/AZD1222, CoronaVac/AZD1222, and AZD1222/BNT162 vaccine regimens demonstrated consistent trends, in which, at the post-boost visit, the levels were significantly higher among adult participants in all vaccine groups (*p* < 0.05) (Table 3) (Supplementary Figure S3). 

### 3.4. Neutralizing Antibody against SARS-CoV-2 Variants of Concern Following Completion of a Primary Series of COVID-19 Vaccination

At the post-boost visit, 89-100% of participants in each vaccine group had detectable NAb-WT, based on an in-house sVNT. Among those receiving CoronaVac/CoronaVac, the proportion of participants with detectable NAb-Alpha (*p* < 0.001) and NAb-Beta (*p* < 0.001), but not for NAb-Delta (*p* = 0.16), were significantly lower than that of NAb-WT. Likewise, among those receiving AZD1222/AZD1222 and CoronaVac/AZD1222, the proportion of detectable NAb-Alpha (*p* < 0.001), NAb-Beta (*p* < 0.001), NAb-Delta (AZD1222/AZD1222: *p* < 0.001; CoronaVac/AZD1222: *p* = 0.01), and NAb-Omicron (*p* < 0.001) were considerably lesser than those of NAb-WT. However, for those receiving AZD1222/BNT162b2 and BNT162b2/BNT162b2, the proportion of participants with detectable neutralizing activity still remained above 95% for NAb-Alpha, NAb-Beta, and NAb-Delta, but substantially dropped to less than 50% for NAb-Omicron which were significantly lower than those of NAb-WT (*p* < 0.001) (Supplementary Table S3). In the stratified analyses by participant’s age group, neutralizing activities against VoCs for adult and elderly participants receiving AZD1222/AZD1222, CoronaVac/AZD1222 and AZD1222/BNT162b2 vaccine regimens demonstrated corresponding patterns (Supplementary Table S3).

**Figure 3:**
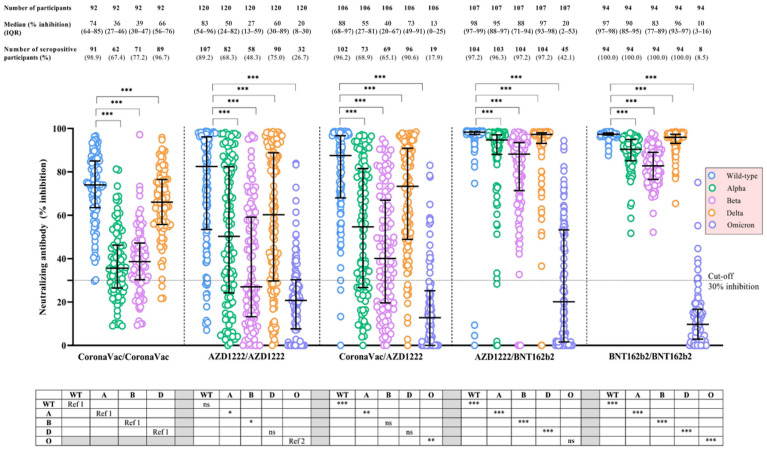
Neutralizing antibody, based on in-house surrogate virus neutralization test, against wild-type SARS-CoV-2 and other circulating variants of concern following the completion of a primary series of COVID-19 vaccination, stratified by the COVID-19 vaccine regimen. Data points are the reciprocals of individual participants. The median values of neutralizing antibody (NAb; in-house surrogate virus neutralization test) and the corresponding interquartile ranges (IQR) against wild-type SARS-CoV-2 and other circulating variants of concern following the completion of a primary series of COVID-19 vaccination are shown as black solid lines. Black dotted lines indicate the cut-off values. The Wilcoxon signed-rank test was performed to compare the medians of NAb between wild-type SARS-CoV-2 and the indicated variant of concern within each COVID-19 vaccine regimen, and the results are shown in the area above the figure. The median regression analysis was performed to compare the medians of NAb to wild-type SARS-CoV-2 or the indicated variant of concern between COVID-19 vaccine regimens, and the results are shown in the table below the figure. The comparisons of NAb-WT, NAb-Alpha, NAb-Beta, and NAb-Delta were conducted between the indicated COVID-19 vaccine regimen and the homologous CoronaVac regimen (the reference vaccine regimen, Ref. 1, whereas the comparisons of NAb-Omicron were conducted between the indicated vaccine regimen and the homologous AZD1222 regimen (the reference vaccine regimen, Ref. 2. * indicates *p* < 0.05; ** indicates *p* < 0.01; *** indicates *p* < 0.001; and ns indicates non-significant for the comparison of NAb between wild-type SARS-CoV-2 and the indicated variant of concern within each COVID-19 vaccine regimen and the comparison of NAb against the indicated variant of concern between vaccine regimens. Abbreviations: A, the SARS-CoV-2 Alpha variant; AZD1222 vaccine, the Oxford-AstraZeneca COVID-19 vaccine; B, the SARS-CoV-2 Beta variant; BNT162b2, Pfizer-BioNTech vaccine; COVID-19, coronavirus disease 2019; D, the SARS-CoV-2 Delta variant; IQR, interquartile range; NAb, neutralizing antibody; NAb-Alpha, neutralizing antibody against Alpha variant; NAb-Beta, neutralizing antibody against Beta variant; NAb-Delta, neutralizing antibody against Delta variant; NAb-O, neutralizing antibody against Omicron variant; NAb-WT, neutralizing antibody against wild-type SARS-CoV-2; O, the SARS-CoV-2 Omicron variant; Ref. 1, the homologous CoronaVac regimen as the reference COVID-19 vaccine regimen for the comparison; Ref. 2, the homologous AZD1222 regimen as the reference COVID-19 vaccine regimen for the comparison; SARS-CoV-2, severe acute respiratory syndrome coronavirus-2.

For each vaccine regimen, the median NAb levels against VoCs, particularly the Omicron strain, at the post-boost visit were significantly attenuated compared with that of NAb-WT (*p* < 0.001) (Table 4) (Figure 3). Similar findings were noted for adult and elderly participants receiving AZD1222/AZD1222, CoronaVac/AZD1222, and AZD1222/BNT162 vaccine regimens (Table 4) (Supplementary Figure S4). Compared across vaccine regimens, the median NAb-Omicron levels were highest among participants receiving AZD1222/BNT162b2 and AZD1222/AZD1222 (20% inhibition), followed by CoronaVac/AZD1222 (13% inhibition) and BNT162b2/BNT162b2 (10% inhibition), respectively (*p* < 0.01) (Table 4) (Figure 3). The patterns of NAb responses against each VoC, stratified by the COVID-19 vaccine regimen and participant’s age group, are demonstrated in Table 4 and Supplementary Figure S4. Notably, the median NAb-Omicron level among adults receiving AZD1222/BNT162 was significantly higher than that of elderly participants (*p* < 0.001), but such a difference was not demonstrated among those receiving AZD1222/AZD1222 (*p* = 0.47) or CoronaVac/AZD1222 vaccine regimens (*p* = 0.85).

### 3.5. Adverse Events Following a Primary Series of COVID-19 Vaccination

No immediate AEs were observed within 30 minutes following each vaccine dose in all regimens. During the seven days after a priming dose, the overall incidence of solicited AEs was 57%, 81%, 57%, 90%, and 94% among participants receiving CoronaVac/CoronaVac, AZD1222/AZD1222, CoronaVac/AZD1222, AZD1222/BNT162b2, and BNT162b2/ BNT162b2 vaccine regimens, respectively. The most common local AE was pain at the injection site, which was greatest among participants in the BNT162b2/BNT162b2 group (93%). Fatigue was the most common systemic AE, and the incidence was greatest among those in the AZD1222/BNT162b2 group (61%) (Table 5). After a booster dose, the overall incidence of solicited AEs was 57%, 71%, 76%, 68%, and 96% among participants receiving CoronaVac/CoronaVac, AZD1222/AZD1222, CoronaVac/AZD1222, AZD1222/BNT162b2, and BNT162b2/BNT162b2 vaccine regimens, respectively. Similar to a priming dose, pain at the injection site was the most common local AE and was greatest among participants in the BNT162b2/BNT162b2 group (93%); whereas fatigue was the most common systemic AE and was greatest among participants in the CoronaVac/AZD1222 group (45%) (Table 5). All reported AEs were mild. The majority occurred within the first three days following vaccination and spontaneously resolved within a few days without requiring any specific treatment. No serious AEs were observed in this study.

## 4. Discussion

In healthy Thai populations, all five primary series of COVID-19 vaccine regimens, including homologous CoronaVac, AZD1222, and BNT162b2, as well as heterologous CoronaVac/AZD1222 and AZD1222/BNT162b2, were well-tolerated and highly immunogenic against wild-type SARS-CoV-2. However, the immunogenicity, both anti-RBD IgG and NAb, against SARS-CoV-2 variants, particularly the Omicron strain, was markedly attenuated. Patterns of vaccine-elicited antibody responses were similar for adult and elderly participants, but higher levels were obtained in adults at the post-boost visit. This study provides important information regarding the immunogenicity and safety profiles of several primary series of COVID-19 vaccine regimens, which could help guide the appropriate primary and alternative vaccine regimens for countries with limited vaccine supplies and restricted access to some vaccine platforms.

In this study, homologous BNT162b2 generated a robust and rapid humoral immune response to wild-type SARS-CoV-2. Compared with the other vaccine regimens, it induced the highest anti-RBD-WT IgG level with a 100% seroconversion at 4 weeks following an initial dose and a 9-fold increase in antibody level following a second dose of vaccine. Similar to a population-based prospective cohort study in Northern Cyprus, the homologous BNT162b2 vaccine regimen induced the highest anti-RBD-WT IgG level and seropositivity in adult (aged < 60 years) and elderly participants (aged *≥* 60 years), followed by the homologous AZD1222 and CoronaVac regimens [17]. Although, in this study, heterologous AZD1222/BNT162b2 elicited a significantly lower anti-RBD-WT IgG level compared to a homologous BNT162b2 regimen, the antibody level at the post-boost visit was considerably higher compared to a homologous AZD1222 regimen, which was consistent with earlier studies in European countries [7,18]. We also noted that the heterologous CoronaVac/AZD1222 vaccine regimen induced significantly higher binding antibodies than either homologous CoronaVac or AZD1222 vaccination, corresponding to previous studies in Thailand [19,20].

Focusing on functional antibodies, the highest vaccine-elicited NAb-WT in this study was observed in the heterologous AZD1222/BNT162b2 vaccine group. Consistent with other findings, vaccination priming with AZD1222 and boosting with BNT162b2 induced a stronger NAb-WT than homologous AZD1222 or BNT162b2 [21,22]. In addition, we noted that heterologous CoronaVac/AZD1222 induced a stronger NAb-WT response than homologous CoronaVac and AZD1222 vaccination, which was similar to the previous study [20]. To date, the mechanism of robust immunogenicity induced by heterologous COVID-19 vaccination is not clearly understood. The possible explanations might be due to the distinct types of host immunity against a specific antigen activated by different vaccine platforms [23] and the curtailment of anti-vector immunity caused by subsequent AZD1222 vaccination [24].

Phylogenetic analysis of SARS-CoV-2 genomes found a substantial number of mutations in the RBD region of SARS-CoV-2 variants. Such mutations considerably impair the binding affinity of vaccine-induced antibodies, resulting in immune evasion of the variants [25,26]. Consistent with previous findings [27,28,29], this study demonstrated that vaccine-elicited NAb levels against important circulating VoCs, particularly the Omicron strain (reduced by 4 to 10-fold), were substantially attenuated for all vaccine regimens compared with those of wild-type SARS-CoV-2. Since all studied COVID-19 vaccine regimens are designed to generate immunity against the spike protein of the wild-type virus, neutralizing capacities against SARS-CoV-2 variants might be impaired [25]. This highlights the need for new COVID-19 booster vaccines, e.g., a bivalent mRNA vaccine that contains mRNA encoding wild-type and Omicron spike proteins to counter the emergence of vaccine-resistant mutations [30].

This study illustrated the poorer vaccine-induced humoral immune responses among elderly participants receiving the homologous AZD1222, and the heterologous CoronaVac/AZD1222 and AZD1222/BNT162b2 vaccine regimens, compared with adult participants. The weakened antibody responses to COVID-19 vaccines in an aging population were previously demonstrated in a number of studies with several vaccine regimens [31,32,33]. The underlying mechanism is immune senescence, which results in a decline in adaptive immunity with increasing age [34]. Thus, elderly populations remain vulnerable to COVID-19 despite primary vaccination, and a booster vaccine is necessary.

All five COVID-19 vaccine regimens are safe and well-tolerated among our participants. Similar to previous studies, transient pain at the injection site and fatigue were the primary solicited local and systemic reactions [35,36]. We noted that an injection site pain was related to the BNT162b2 vaccine, which might be attributed to temporal fasciitis of the deltoid muscle as there is evidence showing a significant increase in fascia thickness without intramuscular echogenicity change in ultrasound images among vaccine recipients [37]. Additionally, we found that fatigue was associated with the AZD1222 vaccine, which might be due to a mild and short-lived increase in circulating inflammatory cytokines in vaccinees [38]. Although no immediate or serious AEs were observed throughout the study period, with a relatively small study cohort, we had limited ability to ascertain rare serious AEs for each vaccine regimen.

This study contains some strengths. We were able to conduct a population-based, head-to-head comparative study to evaluate humoral immunogenicity and reactogenicity of five primary series of COVID-19 vaccine regimens against wild-type SARS-CoV-2 and important circulating VoCs, including the Omicron strain. Nevertheless, there are some limitations. Firstly, since this study is not an RCT, several potential unknown and uncontrollable confounding factors that could bias the study outcomes might not be appropriately adjusted. The studied vaccine regimens relied on vaccine supplies from the Thai government, and eligible populations were determined by the recommendations of the MOPH of Thailand during the study period; therefore, some specific vaccines, e.g., mRNA1273 and Sinopharm BBIBP, as well as some specific populations, e.g., elderly participants in homologous CoronaVac and BNT162b2 vaccine groups, were not evaluated. In addition, the significant difference in age of participants across the five vaccine groups might contribute to the differences in humoral immune responses to vaccination demonstrated in this study. Moreover, while robust antibody responses following primary vaccination have been demonstrated, the durability and trajectory patterns are needed to be monitored. A follow-up study is currently ongoing to demonstrate the antibody decay rates and the importance of booster vaccination. Furthermore, a study of cellular immunity, which plays a vital role in mediating host immune responses to vaccines, is underway. Lastly, since we enrolled only healthy participants, our findings may not be generalizable to individuals with substantial comorbidities or clinical frailty.

## 5. Conclusions

All five primary series of COVID-19 vaccine regimens were well-tolerated and had a favorable safety profile. All regimens elicited robust humoral immune responses against wild-type SARS-CoV-2 but attenuated responses against VoCs, particularly the Omicron strain, among healthy Thai populations. This study provides important evidence for planning an appropriate COVID-19 vaccination program, prioritizing vaccine allocation, and reducing vaccine hesitancy in low-income countries where vaccination coverage remains suboptimal and vaccine supplies are limited.

## Figures and Tables

**Table 1:** Characteristics of study participants, stratified by the COVID-19 vaccine regimen.

Characteristics ^a^	CoronaVac/ CoronaVac	AZD1222/ AZD1222	CoronaVac/ AZD1222	AZD1222/ BNT162b2	BNT162b2/ BNT162b2	*P ^b^*
Number of enrolled participants	332	221	110	128	110	
Age, years	39.0 (30.0–48.0)	62.6 (52.3–68.3)	59.8 (44.0–65.2)	52.3 (30.7–67.5)	28.7 (22.8–36.9)	<0.001
Age category						<0.001
<60 years	332 (100)	73 (33.0)	55 (50.0)	73 (57.0)	110 (100)	
≥60 years	0 (0)	148 (67.0)	55 (50.0)	55 (43.0)	0 (0)	
Sex						0.003
Male	90 (27.1)	81 (36.7)	48 (43.6)	44 (34.4)	27 (24.6)	
Female	242 (72.9)	140 (63.3)	62 (56.4)	84 (65.6)	83 (75.4)	
Body mass index, kg/m^2^	23.1 (20.9–26.0)	24.7 (22.0–27.3)	27.4 (23.7–35.0)	23.2 (20.3–26.2)	22.5 (19.8–26.6)	<0.001
Body mass index category						<0.001
Underweight	20 (6.0)	10 (4.5)	2 (1.8)	8 (6.3)	14 (12.7)	
Normal weight	201 (60.6)	108 (48.9)	40 (36.4)	78 (60.9)	56 (50.9)	
Overweight	80 (24.1)	75 (33.9)	36 (32.7)	34 (26.6)	28 (25.5)	
Obese	31 (9.3)	28 (12.7)	32 (29.1)	8 (6.2)	12 (10.9)	
Cigarette smoking						<0.001
Never smoked	326 (98.2)	197 (89.1)	88 (80.0)	120 (93.8)	103 (93.6)	
Ever smoked (current and former)	6 (1.8)	24 (10.9)	22 (20.0)	8 (6.2)	7 (6.4)	
Alcohol consumption						<0.001
Never drunk	287 (86.5)	168 (76.0)	71 (64.6)	100 (78.1)	87 (79.1)	
Ever drunk (current and former)	45 (13.5)	53 (24.0)	39 (35.4)	28 (21.9)	23 (20.9)	

Abbreviations: AZD1222 vaccine, the Oxford-AstraZeneca COVID-19 vaccine; BNT162b2; Pfizer-BioNTech vaccine; COVID-19, coronavirus disease 2019; ^a^ Data were presented as number (percentage) for categorical data and median (interquartile range) for continuous data; ^b^ The comparisons across five COVID-19 vaccine regimens were performed by the Chi-squared test or the Fisher’s exact test (if less than five observations) for categorical data and by the Wilcoxon rank-sum test for continuous data.

**Table 2:** The geometric mean concentrations of anti-SARS-CoV-2 receptor-binding domain immunoglobulin G against wild-type SARS-CoV-2 following a primary series of COVID-19 vaccination, stratified by the COVID-19 vaccine regimen.

Study Participant and Study Visit	CoronaVac/CoronaVac			AZD1222/AZD1222			CoronaVac/AZD1222			AZD1222/BNT162b2			BNT162b2/BNT162b2		
	GMC of Anti- RBD-WT IgG (95% CI, BAU/mL)	GMR ^a^ (95% CI)	*P ^a^*	GMC of Anti- RBD-WT IgG (95% CI, BAU/mL)	GMR ^a^ (95% CI)	*P ^a^*	GMC of Anti- RBD-WT IgG (95% CI, BAU/mL)	GMR ^a^ (95% CI)	*P ^a^*	GMC of Anti- RBD-WT IgG (95% CI, BAU/mL)	GMR ^a^ (95% CI)	*P ^a^*	GMC of Anti- RBD-WT IgG (95% CI, BAU/mL)	GMR ^a^ (95% CI)	*P ^a^*
***All participants***															
Pre-prime visit	0.3 (0.2–0.3)			0.5 (0.4–0.7)			0.6 (0.3–1.0)			0.4 (0.3–0.5)			1.0 (0.5–2.0)		
Post-prime visit	8.4 (7.4–9.5)	29.1 (23.9–35.4)	<0.001	58.8 (50.0–69.2)	114.1 (82.9–157.1)	<0.001	6.7 (5.1–8.9)	12.5 (7.6–20.6)	<0.001	73.6 (58.1–93.1)	211.0 (138.0–322.6)	<0.001	191.2 (164.3–222.4)	193.9 (122.7–306.4)	<0.001
Post-boost visit	122.2 (111.4–134.0)	14.7 (13.1–16.6)	<0.001	179.9 * (157.3–205.9)	3.0 (2.6–3.6)	<0.001	488.6 * (410.4–581.7)	74.8 (61.3–91.3)	<0.001	1156.6 * (1009.2–1325.7)	15.9 (13.2–19.1)	<0.001	1697.6 * (1510.9–1907.4)	9.0 (8.1–10.1)	<0.001
***Adults aged < 60 years old***															
Pre-prime visit	0.3 (0.2–0.3)			0.6 (0.3–1.0)			0.8 (0.4–1.8)			0.3 (0.2–0.4)			1.0 (0.5–2.0)		
Post-prime visit	8.4 (7.4–9.5)	29.1 (23.9–35.4)	<0.001	72.6 (54.6–96.5)	131.3 (80.5–214.2)	<0.001	9.7 (6.9–13.7)	11.7 (5.5–24.9)	<0.001	100.3 (79.9–125.9)	330.0 (224.5–485.1)	<0.001	191.2 (164.3–222.4)	193.9 (122.7–306.4)	<0.001
Post-boost visit	122.2 (111.4–134.0)	14.7 (13.1–16.6)	<0.001	258.3 * (207.2–321.8)	3.5 (2.6–4.8)	<0.001	566.4 * (435.0–737.6)	58.5 (46.4–73.8)	<0.001	1279.1 * (1111.8–1471.5)	12.8 (83.4–15.4)	<0.001	1697.6 * (1510.9–1907.4)	9.0 (8.1–10.1)	<0.001
***Elderly aged ≥ 60 years old***															
Pre-prime visit	-	-	-	0.5 (0.3–0.7)			0.4 (0.2–0.9)			1.1 (0.4–3.2)			-	-	-
Post-prime visit	-	-	-	53.0 (43.5–64.5)	109.5 (72.3–165.8)	<0.001	4.6 (3.0–7.1)	10.0 (5.5–18.3)	<0.001	46.6 (29.3–73.4)	90.7 (35.6–231.1)	<0.001	-	-	-
Post-boost visit	-	-	-	150.0 (127.4–176.6)	2.8 (2.3–3.4)	<0.001	421.4 ^†^ (335.0–530.1)	96.6 (80.9–131.6)	<0.001	989.3 ^†^ (752.6–1300.5)	22.4 (15.7–31.9)	<0.001	-	-	-

Abbreviations: AZD1222 vaccine, the Oxford-AstraZeneca COVID-19 vaccine; BAU, binding antibody unit; BNT162b2, Pfizer-BioNTech vaccine; COVID-19, coronavirus disease 2019; GMC, geometric mean concentration; GMR, geometric mean ratio; IgG, immunoglobulin G; RBD, receptor-binding domain; SARS-CoV-2, severe acute respiratory syndrome coronavirus-2; WT, wild-type SARS-CoV-2; 95% CI, 95% confidence interval; ^a^ The mixed-effects log-linear regression analysis was performed to compare the geometric mean concentrations of anti-SARS-CoV-2 receptor-binding domain immunoglobulin G against wild-type SARS-CoV-2 between the indicated visit and the previous visit within each COVID-19 vaccine regimen.; * Indicates a significant difference (*p* < 0.001) of the geometric mean concentration of anti-SARS-CoV-2 receptor-binding domain immunoglobulin G against wild-type SARS-CoV-2 after completion of the primary series of an indicated COVID-19 vaccination compared with the homologous CoronaVac vaccine regimen, evaluated by the log-linear regression analysis; ^†^ Indicates a significant difference (*p* < 0.001) of the geometric mean concentration of anti-SARS-CoV-2 receptor-binding domain immunoglobulin G against wild-type SARS-CoV-2 after completion of the primary series of an indicated COVID-19 vaccination compared with the homologous AZD1222 vaccine regimen, evaluated by the log-linear regression analysis.

**Table 3:** The median levels of neutralizing antibodies against wild-type SARS-CoV-2 following a primary series of COVID-19 vaccination, stratified by the COVID-19 vaccine regimen.

Study Participant and Study Visit	CoronaVac/CoronaVac			AZD1222/AZD1222			CoronaVac/AZD1222			AZD1222/BNT162b2			BNT162b2/BNT162b2		
	Median of NAb-WT (IQR, % Inhibition)	Median Difference ^a^ (IQR, % Inhibition)	*P ^a^*	Median of NAb-WT (IQR, % Inhibition)	Median Difference ^a^ (IQR, % Inhibition)	*P ^a^*	Median of NAb-WT (IQR, % Inhibition)	Median Difference ^a^ (IQR, % Inhibition)	*P ^a^*	Median of NAb-WT (IQR, % Inhibition)	Median Difference ^a^ (IQR, % Inhibition)	*P ^a^*	Median of NAb-WT (IQR, % Inhibition)	Median Difference ^a^ (IQR, % Inhibition)	*P ^a^*
***All participants***															
Pre-prime visit	0			0			0			0			0		
Post-prime visit	2.7 (0–10.4)	2.4 (0–10.3)	<0.001	36.1 (16.6–58.6)	36.1 (36.1–58.2)	<0.001	0 (0.0–3.8)	0 (0–3.8)	<0.001	38.1 (17.7–63.9)	37.6 (17.4–62.8)	<0.001	58.8 (39.8–71.8)	58.8 (37.5–71.8)	<0.001
Post-boost visit	59.9 (42.5–75.2)	51.6 (35.6–68.3)	<0.001	76.7 * (54.9–92.3)	31.7 (9.5–53.3)	<0.001	91.9 * (76.6–97.6)	85.9 (72.3–93.4)	<0.001	98.8 * (97.6–99.2)	59.4 (33.4–77.1)	<0.001	98.4 * (96.5–99.1)	40.5 (26.8–58.4)	<0.001
***Adults aged < 60 years old***															
Pre-prime visit	0			0			0			0			0		
Post-prime visit	2.7 (0–10.4)	2.4 (0–10.3)	<0.001	45.6 (19.9–64.7)	43.8 (17.2–64.7)	<0.001	0 (0–4.9)	0 (0–4.9)	<0.001	47.0 (27.5–68.2)	45.8 (26.8–68.2)	<0.001	58.8 (39.8–71.8)	58.8 (37.5–71.8)	<0.001
Post-boost visit	59.9 (42.5–75.2)	51.6 (35.6–68.3)	<0.001	85.4 * (67.0–94.7)	33.1 (12.7–60.5)	<0.001	94.0 * (83.5–98.6)	86.4 (77.9–94.0)	<0.001	99.1 * (98.4–99.4)	51.6 (30.9–69.0)	<0.001	98.4 * (96.5–99.1)	40.5 (26.8–58.4)	<0.001
***Elderly aged ≥ 60 years old***															
Pre-prime visit	-	-	-	0			0			0			-	-	-
Post-prime visit	-	-	-	34.7 (15.5–53.7)	34.4 (15.5–53.5)	<0.001	0 (0.0–3.8)	0 (0–3.8)	<0.001	19.9 (5.4–52.0)	19.9 (5.4–52.0)	<0.001	-	-	-
Post-boost visit	-	-	-	73.3 (47.2–89.9)	31.0 (9.1–52.5)	<0.001	89.9 ^†^ (70.2–95.0)	81.2 (65.1–91.9)	<0.001	98.0 ^†^ (94.9–99.0)	72.0 (39.2–80.8)	<0.001	-	-	-

Abbreviations: AZD1222 vaccine, the Oxford-AstraZeneca COVID-19 vaccine; BNT162b2, Pfizer-BioNTech vaccine; COVID-19, coronavirus disease 2019; IQR, interquartile range; NAb, neutralizing antibody; SARS-CoV-2, severe acute respiratory syndrome coronavirus-2; WT, wild-type SARS-CoV-2; ^a^ The Wilcoxon signed-rank test was performed to compare the medians of neutralizing antibody against wild-type SARS-CoV-2 between the indicated visit and the previous visit within each COVID-19 vaccine regimen; * Indicates a significant difference (*p* < 0.001) of the median of neutralizing antibodies against wild-type SARS-CoV-2 after completion of the primary series of an indicated COVID-19 vaccination, compared with the homologous CoronaVac vaccine regimen, evaluated by the median regression analysis; ^†^ Indicates a significant difference (*p* < 0.01) of the median of neutralizing antibodies against wild-type SARS-CoV-2 after completion of the primary series of an indicated COVID-19 vaccination, compared with the homologous AZD1222 vaccine regimen, evaluated by the median regression analysis.

**Table 4:** The median of neutralizing antibodies, based on the in-house surrogate virus neutralization test, against wild-type and other circulating variants of concern of SARS-CoV-2 following the completion of a primary series of COVID-19 vaccination, stratified by the COVID-19 vaccine regimen.

Study Participant and SARS-CoV-2 Variant	CoronaVac/CoronaVac			AZD1222/AZD1222			CoronaVac/AZD1222			AZD1222/BNT162b2			BNT162b2/BNT162b2		
	Median of NAb-VoC (IQR, % Inhibition)	Median Difference ^a^ (IQR, % Inhibition)	*P ^a^*	Median of NAb-VoC (IQR, % Inhibition)	Median Difference ^a^ (IQR, % Inhibition)	*P ^a^*	Median of NAb-VoC (IQR, % Inhibition)	Median Difference ^a^ (IQR, % Inhibition)	*P ^a^*	Median of NAb-VoC (IQR, % Inhibition)	Median Difference ^a^ (IQR, % Inhibition)	*P ^a^*	Median of NAb-VoC (IQR, % Inhibition)	Median Difference ^a^ (IQR, % Inhibition)	*P ^a^*
***All participants***															
Wild-type	74.0 (63.6–85.0)	Ref	Ref	82.5 (53.7–96.0)	Ref	Ref	87.5 * (68.2–96.7)	Ref	Ref	98.3 * (97.3–98.5)	Ref	Ref	97.3 * (96.9–97.9)	Ref	Ref
Alpha	35.6 (26.6–46.1)	−35.4 (−39.8 to –31.1)	<0.001	50.3 * (24.3–82.3)	-19.8 (−32.0 to –9.6)	<0.001	54.6 * (26.7–81.4)	−25.0 (−41.6 to–12.6)	<0.001	94.7 * (88.1–97.1)	−3.2 (−7.9 to –1.4)	<0.001	90.4 * (85.2–94.9)	−6.8 (−11.8 to –3.2)	<0.001
Beta	38.6 (30.4–47.0)	−34.2 (−41.2 to –24.7)	<0.001	27.0 * (13.4–58.8)	−35.5 (−47.9 to –22.5)	<0.001	40.1 (19.6–66.5)	−38.4 (−49.1 to 22.0)	<0.001	88.2* (71.4–93.5)	−9.8 (−22.9 to –4.5)	<0.001	82.8 * (76.6–88.8)	−14.1 (−20.4 to –8.6)	<0.001
Delta	66.1 (55.8–76.4)	−7.5 (−11.3 to –3.2)	<0.001	60.2 (29.9–88.5)	−11.4 (−23.9 to –3.9)	<0.001	73.3 (48.9–90.9)	−8.1 (−19.1 to –2.8)	<0.001	97.3 * (93.1–98.0)	−1.0 (−4.2 to –0.5)	<0.001	96.0 * (93.2–97.3)	−1.5 (−3.8 to –0.4)	<0.001
Omicron	-	-	-	20.4 (7.9–30.3)	−54.3 (−72.0 to –33.0)	<0.001	12.8 ^†^ (0–24.8)	−64.3 (−80.2 to –51.2)	<0.001	20.4 (1.6–53.3)	−74.7 (−92.9 to –44.0)	<0.001	9.7 ^†^ (3.0–16.3)	−86.4 (−94.0 to –80.7)	<0.001
***Adults aged < 60 years old***															
Wild-type	74.0 (63.6–85.0)	−35.4 (−39.8 to –31.1)	<0.001	90.7 * (63.8–97.5)	Ref	Ref	93.8 * (69.3–97.9)	Ref	Ref	98.5 * (98.2–98.6)	Ref	Ref	97.3 * (96.9–97.9)	Ref	Ref
Alpha	35.6 (26.6–46.1)	−34.2 (−−41.2 to –24.7)	<0.001	68.6 * (27.9–87.2)	−17.2 (−30.1 to –8.2)	<0.001	66.6 * (33.8–90.3)	−16.4 (−34.6 to –7.1)	<0.001	96.9 * (93.4–97.6)	−1.8 (−4.1 to –1.02)	<0.001	90.4 * (85.2–94.9)	−6.8 (−11.8 to –3.2)	<0.001
Beta	38.6 (30.4–47.0)	−7.5 (−11.3 to –3.2)	<0.001	48.5 (20.7–70.8)	−32.0 (−41.8 to –20.4)	<0.001	46.6 (27.1–70.9)	−32.2 (−49.0 to –16.0)	<0.001	91.5 * (82.1–95.5)	−6.6 (−12.7 to –3.2)	<0.001	82.8 * (76.6–88.8)	−14.1 (−20.4 to –8.6)	<0.001
Delta	66.1 (55.8–76.4)	−35.4 (−39.8 to –31.1)	<0.001	80.2 * (38.5–93.2)	−7.8 (−23.4 to –3.1)	<0.001	83.6 * (56.0–95.0)	−4.9 (−10.4 to –1.4)	<0.001	97.7 * (96.3–98.1)	−0.9 (−1.8 to –5.4)	<0.001	96.0 * (93.2–97.3)	−1.5 (−3.8 to –0.4)	<0.001
Omicron	-	-	-	21.1 (0.2–30.2)	−57.0 (−81.0 to –37.8)	<0.001	9.7 (0–26.3)	−65.4 (−82.3 to –51.9)	<0.001	51.5 ^†^ (25.1–61.6)	−45.8 (−69.2 to –34.7)	<0.001	9.7 ^†^ (3.0–16.3)	−86.4 (−94.0 to –80.7)	<0.001
***Elderly aged ≥ 60 years old***															
Wild-type	-	-	-	74.9 (45.3–92.9)	Ref	Ref	79.7 (67.2–93.6)	Ref	Ref	98.0 ^†^ (96.3–98.3)	Ref	Ref	-	-	-
Alpha	-	-	-	42.3 (14.8–66.9)	−24.8 (−32.7 to –11.6)	<0.001	38.6 (26.6–70.1)	−28.2 (−41.8 to –21.4)	<0.001	90.3 ^†^ (68.7–94.7)	−7.6 (−24.8 to –3.2)	<0.001	-	-	-
Beta	-	-	-	18.0 (7.9–44.0)	–39.6 (−54.2 to –29.0)	<0.001	34.3^†^ (19.6–54.4)	−42.6 (−49.1 to –25.4)	<0.001	78.8 ^†^ (58.3–90.9)	−16.2 (−33.7 to –6.7)	<0.001	-	-	-
Delta	-	-	-	44.8 (28.2–83.9)	−13.9 (−25.7 to –6.2)	<0.001	59.8 (44.0–85.8)	−13.0 (−22.2 to –5.5)	<0.001	93.9 ^†^ (77.0–97.7)	−2.9 (−14.7 to –0.5)	<0.001	-	-	-
Omicron	-	-	-	20.6 (14.2–30.3)	−50.3 (−62.7 to –28.4)	<0.001	13.3 ^†^ (1.1–23.0)	−59.1 (−76.6 to –50.4)	<0.001	1.8 ^†^ (0–7.7)	−92.9 (−96.3 to –83.0)	<0.001	-	-	-

Abbreviations: AZD1222 vaccine, the Oxford-AstraZeneca COVID-19 vaccine; BNT162b2, Pfizer-BioNTech vaccine; COVID-19, coronavirus disease 2019; IQR, interquartile range; NAb, neutralizing antibody; Ref, reference; SARS-CoV-2, severe acute respiratory syndrome coronavirus-2; VoC, variant of concern; ^a^ The Wilcoxon signed-rank test was performed to compare the medians of neutralizing antibody, based on the in-house surrogate virus neutralization test, between wild-type SARS-CoV-2 and the indicated variant of concern within each COVID-19 vaccine regimen; * Indicates a significant difference (*p* < 0.05) of the median of neutralizing antibodies, based on the in-house surrogate virus neutralization test, against wild-type and other circulating variants of concern of SARS-CoV-2 after the completion of the primary series of an indicated COVID-19 vaccination compared with the homologous CoronaVac vaccine regimen, evaluated by the median regression analysis; ^†^ Indicates a significant difference (*p* < 0.05) of the median of neutralizing antibodies, based on the in-house surrogate virus neutralization test, against wild-type and other circulating variants of concern of SARS-CoV-2 after the completion of the primary series of an indicated COVID-19 vaccination compared with the homologous AZD1222 vaccine regimen, evaluated by the median regression analysis.

**Table 5:** The solicited local and systemic adverse events within seven days following a primary series of COVID-19 vaccination, stratified by the COVID-19 vaccine regimen.

Adverse Events ^a^	CoronaVac/ CoronaVac	AZD1222/ AZD1222	CoronaVac/ AZD1222	AZD1222/ BNT162b2	BNT162b2/ BNT162b2	*P ^b^*
**First dose of vaccine**	(*n* = 286)	(*n* = 221)	(*n* = 109)	(*n* = 100)	(*n* = 83)	
Overall adverse events	164 (57.3)	179 (81.0)	62 (56.9)	90 (90.0)	78 (94.0)	<0.001
***Local adverse events***						
Pain	116 (40.6)	132 (59.7)	28 (25.7)	74 (74.0)	77 (92.8)	<0.001
Swelling	6 (2.1)	29 (13.1)	3 (2.8)	11 (11.0)	18 (21.7)	<0.001
Erythema	4 (1.4)	14 (6.3)	5 (4.6)	10 (10.0)	4 (4.8)	0.002
***Systemic adverse events***						
Fatigue	87 (30.4)	94 (42.5)	41 (37.6)	61 (61.0)	33 (39.8)	<0.001
Headache	50 (17.5)	72 (32.6)	20 (18.4)	43 (43.0)	16 (19.3)	<0.001
Fever	11 (3.9)	64 (29.0)	13 (11.9)	52 (52.0)	14 (16.9)	<0.001
Paresthesia	10 (3.5)	12 (5.4)	6 (5.5)	3 (3.0)	2 (2.4)	0.65
Dizziness	5 (1.8)	7 (3.2)	4 (3.7)	5 (5.0)	0 (0)	0.16
**Second dose of vaccine**	(*n* = 286)	(*n* = 209)	(*n* = 100)	(*n* = 120)	(*n* = 76)	
Overall adverse events	164 (57.3)	149 (71.3)	76 (76.0)	82 (68.3)	73 (96.1)	<0.001
***Local adverse events***						
Pain	139 (48.6)	100 (47.9)	60 (60.0)	74 (61.7)	71 (93.4)	<0.001
Swelling	7 (2.5)	20 (9.6)	14 (14.0)	21 (17.5)	27 (35.5)	<0.001
Erythema	4 (1.4)	19 (9.1)	5 (5.0)	11 (9.2)	11 (14.5)	<0.001
***Systemic adverse events***						
Fatigue	77 (26.9)	61 (29.2)	45 (45.0)	49 (40.8)	32 (42.1)	0.001
Headache	41 (14.3)	53 (25.4)	42 (42.0)	27 (22.5)	33 (43.4)	<0.001
Fever	11 (3.9)	32 (15.3)	41 (41.0)	32 (26.7)	24 (31.6)	<0.001
Paresthesia	12 (4.2)	5 (2.4)	8 (8.0)	5 (4.2)	2 (2.6)	0.24
Dizziness	3 (1.1)	6 (2.9)	4 (4.0)	1 (0.8)	3 (4.0)	0.14

Abbreviations: AZD1222 vaccine, the Oxford-AstraZeneca COVID-19 vaccine; BNT162b2, Pfizer-BioNTech vaccine; COVID-19, coronavirus disease 2019; ^a^ Data were presented as number (percentage); ^b^ The comparison of adverse events between groups of study participants was performed using the Chi-squared test or the Fisher’s exact test (if less than 5 observations).

## Data Availability

The data that were used and/or analyzed are available upon request from the corresponding author.

## Supplementary Materials

The following supporting information can be downloaded at: https://www.mdpi.com/article/10.3390/vaccines11030564/s1. Figure S1: Diagram of study participants; Figure S2: Anti-SARS-CoV-2 receptor-binding domain immunoglobulin G against wild-type SARS-CoV-2 following a primary series of COVID-19 vaccination, stratified by the COVID-19 vaccine regimen and participant’s age group; Figure S3: Neutralizing antibody against wild-type SARS-CoV-2 following a primary series of COVID-19 vaccination, stratified by the COVID-19 vaccine regimen and participant’s age group; Figure S4: Neutralizing antibody, based on the in-house surrogate virus neutralization test, against wild-type SARS-CoV-2 and other circulating variants of concern following the completion of a primary series of COVID-19 vaccination, stratified by the COVID-19 vaccine regimen and participant’s age group. Table S1: The proportion of seropositive participants based on anti-SARS-CoV-2 receptor-binding domain immunoglobulin G against wild-type SARS-CoV-2 following a primary series of COVID-19 vaccination, stratified by the COVID-19 vaccine regimen; Table S2: The proportion of participants with positive neutralizing activity against wild-type SARS-CoV-2 following a primary series of COVID-19 vaccination, stratified by the COVID-19 vaccine regimen; Table S3: The proportion of participants with positive neutralizing activity, based on the in-house surrogate virus neutralization test, against wild-type and other circulating variants of concern of SARS-CoV-2 following the completion of a primary series of COVID-19 vaccination, stratified by the COVID-19 vaccine regimen.

## References

[1] Zheng C., Shao W., Chen X., Zhang B., Wang G., Zhang W. (2022). Real-world effectiveness of COVID-19 vaccines: A literature review and meta-analysis. Int. J. Infect. Dis..

[2] Liu H., Zhang J., Cai J., Deng X., Peng C., Chen X., Yang J., Wu Q., Chen X., Chen Z. (2021). Herd immunity induced by COVID-19 vaccination programs and suppression of epidemics caused by the SARS-CoV-2 Delta variant in China. medRxiv.

[3] MacIntyre C.R., Costantino V., Trent M. (2022). Modelling of COVID-19 vaccination strategies and herd immunity, in scenarios of limited and full vaccine supply in NSW, Australia. Vaccine.

[4] Basta N.E., Moodie E.M.M., the VIPER (Vaccines, Infectious disease Prevention, and Epidemiology Research) Group COVID-19 Vaccine Development and Approvals Tracker Team COVID-19 Vaccine Development and Approvals Tracker. https://covid19.trackvaccines.org/vaccines/approved/.

[5] Our World in Data. Coronavirus (COVID-19) Vaccinations. https://ourworldindata.org/covid-vaccinations.

[6] Borobia A.M., Carcas A.J., Pérez-Olmeda M., Castaño L., Bertran M.J., García-Pérez J., Campins M., Portolés A., González-Pérez M., Morales M.T.G. (2021). Immunogenicity and reactogenicity of BNT162b2 booster in ChAdOx1-S-primed participants (CombiVacS): A multicentre, open-label, randomised, controlled, phase 2 trial. Lancet.

[7] Liu X., Shaw R.H., Stuart A.S.V., Greenland M., Aley P.K., Andrews N.J., Cameron J.C., Charlton S., Clutterbuck E.A., Collins A.M. (2021). Safety and immunogenicity of heterologous versus homologous prime-boost schedules with an adenoviral vectored and mRNA COVID-19 vaccine (Com-COV): A single-blind, randomised, non-inferiority trial. Lancet.

[8] Atmar R.L., Lyke K.E., Deming M.E., Jackson L.A., Branche A.R., El Sahly H.M., Rostad C.A., Martin J.M., Johnston C., Rupp R.E. (2021). Heterologous SARS-CoV-2 booster vaccinations—Preliminary report. medRxiv.

[9] Gilbert P.B., Montefiori D.C., McDermott A.B., Fong Y., Benkeser D., Deng W., Zhou H., Houchens C.R., Martins K., Jayashankar L. (2022). Immune correlates analysis of the mRNA-1273 COVID-19 vaccine efficacy clinical trial. Science.

[10] Khoury D.S., Cromer D., Reynaldi A., Schlub T.E., Wheatley A.K., Juno J.A., Subbarao K., Kent S.J., Triccas J.A., Davenport M.P. (2021). Neutralizing antibody levels are highly predictive of immune protection from symptomatic SARS-CoV-2 infection. Nat. Med..

[11] Zhang Z., Mateus J., Coelho C.H., Dan J.M., Moderbacher C.R., Gálvez R.I., Cortes F.H., Grifoni A., Tarke A., Chang J. (2022). Humoral and cellular immune memory to four COVID-19 vaccines. Cell.

[12] Dodd R.H., Pickles K., Nickel B., Cvejic E., Ayre J., Batcup C., Bonner C., Copp T., Cornell S., Dakin T. (2021). Concerns and motivations about COVID-19 vaccination. Lancet Infect. Dis..

[13] Department of Disease Control, Ministry of Public Health Guidelines for COVID-19 Vaccination in Thailand. https://ddc.moph.go.th/vaccine-covid19/guidelines.

[14] Department of Disease Control, Ministry of Public Health Daily Report of COVID-19 Vaccine in Thailand. https://ddc.moph.go.th/vaccine-covid19/diaryReport.

[15] World Health Organization WHO Coronavirus (COVID-19) Dashboard—Thailand. https://covid19.who.int/region/searo/country/th.

[16] Tan C.W., Chia W.N., Qin X., Liu P., Chen M.I.C., Tiu C., Hu Z., Chen V.C.W., Young B.E., Sia W.R. (2020). A SARS-CoV-2 surrogate virus neutralization test based on antibody-mediated blockage of ACE2-spike protein-protein interaction. Nat. Biotechnol..

[17] Barin B., Kasap U., Selçuk F., Volkan E., Uluçkan Ö. (2022). Comparison of SARS-CoV-2 anti-spike receptor binding domain IgG antibody responses after CoronaVac, BNT162b2, ChAdOx1 COVID-19 vaccines, and a single booster dose: A prospective, longitudinal population-based study. Lancet Microbe.

[18] Schmidt T., Klemis V., Schub D., Mihm J., Hielscher F., Marx S., Abu-Omar A., Ziegler L., Guckelmus C., Urschel R. (2021). Immunogenicity and reactogenicity of heterologous ChAdOx1 nCoV-19/mRNA vaccination. Nat. Med..

[19] Mahasirimongkol S., Khunphon A., Kwangsukstid O., Sapsutthipas S., Wichaidit M., Rojanawiwat A., Wichuckchinda N., Puangtubtim W., Pimpapai W., Soonthorncharttrawat S. (2022). The pilot study of immunogenicity and adverse events of a COVID-19 vaccine regimen: Priming with inactivated whole SARS-CoV-2 vaccine (CoronaVac) and boosting with the adenoviral vector (ChAdOx1 nCoV-19) vaccine. Vaccines.

[20] Wanlapakorn N., Suntronwong N., Phowatthanasathian H., Yorsaeng R., Vichaiwattana P., Thongmee T., Auphimai C., Srimuan D., Thatsanatorn T., Assawakosri S. (2022). Safety and immunogenicity of heterologous and homologous inactivated and adenoviral-vectored COVID-19 vaccine regimens in healthy adults: A prospective cohort study. Hum. Vaccines Immunother..

[21] Barros-Martins J., Hammerschmidt S.I., Cossmann A., Odak I., Stankov M.V., Ramos G.M., Dopfer-Jablonka A., Heidemann A., Ritter C., Friedrichsen M. (2021). Immune responses against SARS-CoV-2 variants after heterologous and homologous ChAdOx1 nCoV-19/BNT162b2 vaccination. Nat. Med..

[22] Pozzetto B., Legros V., Djebali S., Barateau V., Guibert N., Villard M., Peyrot L., Allatif O., Fassier J.B., Massardier-Pilonchéry A. (2021). Immunogenicity and efficacy of heterologous ChAdOx1-BNT162b2 vaccination. Nature.

[23] Kardani K., Bolhassani A., Shahbazi S. (2016). Prime-boost vaccine strategy against viral infections: Mechanisms and benefits. Vaccine.

[24] Kunal S., Sakthivel P., Gupta N., Ish P. (2021). Mix and match COVID-19 vaccines: Potential benefit and perspective from India. Postgrad. Med. J..

[25] Mistry P., Barmania F., Mellet J., Peta K., Strydom A., Viljoen I.M., James W., Gordon S., Pepper M.S. (2022). SARS-CoV-2 Variants, Vaccines, and Host Immunity. Front. Immunol..

[26] Ao D., Lan T., He X., Liu J., Chen L., Baptista-Hon D.T., Zhang K., Wei X. (2022). SARS-CoV-2 Omicron variant: Immune escape and vaccine development. MedComm.

[27] Cheng S.M.S., Mok C.K.P., Leung Y.W.Y., Ng S.S., Chan K.C.K., Ko F.W., Chen C., Yiu K., Lam B.H.S., Lau E.H.Y. (2022). Neutralizing antibodies against the SARS-CoV-2 Omicron variant BA.1 following homologous and heterologous CoronaVac or BNT162b2 vaccination. Nat. Med..

[28] Niyomnaitham S., Toh Z.Q., Wongprompitak P., Jansarikit L., Srisutthisamphan K., Sapsutthipas S., Jantraphakorn Y., Mingngamsup N., Licciardi P.V., Chokephaibulkit K. (2022). Immunogenicity and reactogenicity against the SARS-CoV-2 variants following heterologous primary series involving CoronaVac, ChAdox1 nCov-19 and BNT162b2 plus BNT162b2 booster vaccination: An open-label randomized study in healthy Thai adults. Hum. Vaccines Immunother..

[29] Pascuale C.A., Varese A., Ojeda D.S., Pasinovich M.E., Lopez L., Rossi A.H., Rodriguez P.E., Miglietta E.A., Group L.S., Mazzitelli I. (2022). Immunogenicity and reactogenicity of heterologous immunization against SARS CoV-2 using Sputnik V, ChAdOx1-S, BBIBP-CorV, Ad5-nCoV, and mRNA-1273. Cell Rep. Med..

[30] Chalkias S., Harper C., Vrbicky K., Walsh S.R., Essink B., Brosz A., McGhee N., Tomassini J.E., Chen X., Chang Y. (2022). A bivalent Omicron-containing booster vaccine against Covid-19. N. Engl. J. Med..

[31] Asano M., Okada H., Itoh Y., Hirata H., Ishikawa K., Yoshida E., Matsui A., Kelly E.J., Shoemaker K., Olsson U. (2022). Immunogenicity and safety of AZD1222 (ChAdOx1 nCoV-19) against SARS-CoV-2 in Japan: A double-blind, randomized controlled phase 1/2 trial. Int. J. Infect. Dis..

[32] Müller L., Andrée M., Moskorz W., Drexler I., Walotka L., Grothmann R., Ptok J., Hillebrandt J., Ritchie A., Rabl D. (2021). Age-dependent immune response to the Biontech/Pfizer BNT162b2 coronavirus disease 2019 vaccination. Clin. Infect. Dis..

[33] Kang Y.M., Minn D., Lim J., Lee K.D., Jo D.H., Choe K.W., Kim M.J., Kim J.M., Kim K.N. (2021). Comparison of antibody response elicited by ChAdOx1 and BNT162b2 COVID-19 vaccine. J. Korean Med. Sci..

[34] Gruver A.L., Hudson L.L., Sempowski G.D. (2007). Immunosenescence of ageing. J. Pathol..

[35] Kadali R.A.K., Janagama R., Peruru S., Malayala S.V. (2021). Side effects of BNT162b2 mRNA COVID-19 vaccine: A randomized, cross-sectional study with detailed self-reported symptoms from healthcare workers. Int. J. Infect. Dis..

[36] Folegatti P.M., Ewer K.J., Aley P.K., Angus B., Becker S., Belij-Rammerstorfer S., Bellamy D., Bibi S., Bittaye M., Clutterbuck E.A. (2020). Safety and immunogenicity of the ChAdOx1 nCoV-19 vaccine against SARS-CoV-2: A preliminary report of a phase 1/2, single-blind, randomised controlled trial. Lancet.

[37] Tomita K., Okada S., Sugihara S., Ikeuchi T., Touge H., Hasegawa J., Yamasaki A. (2021). Physical characteristics of injection site pain after COVID-19 mRNA BNT162b2 vaccination. Yonago Acta Med..

[38] Hervé C., Laupèze B., Giudice G.D., Didierlaurent A.M., Silva F.T.D. (2019). The how’s and what’s of vaccine reactogenicity. NPJ Vaccines.

