# Supplementary material for: Comparison of Immunogenicity and Reactogenicity of Five Primary Series of COVID-19 Vaccine Regimens against Circulating SARS-CoV-2 Variants of Concern among Healthy Thai Populations
Source: Vaccines (Basel). 2023 Mar 1;11(3):564. doi: 10.3390/vaccines11030564 (PMC10051392; doi:10.3390/vaccines11030564)
Supplement: Supplementary file 1 [file vaccines-11-00564-s001.zip › Supplementary_Figures.pdf]

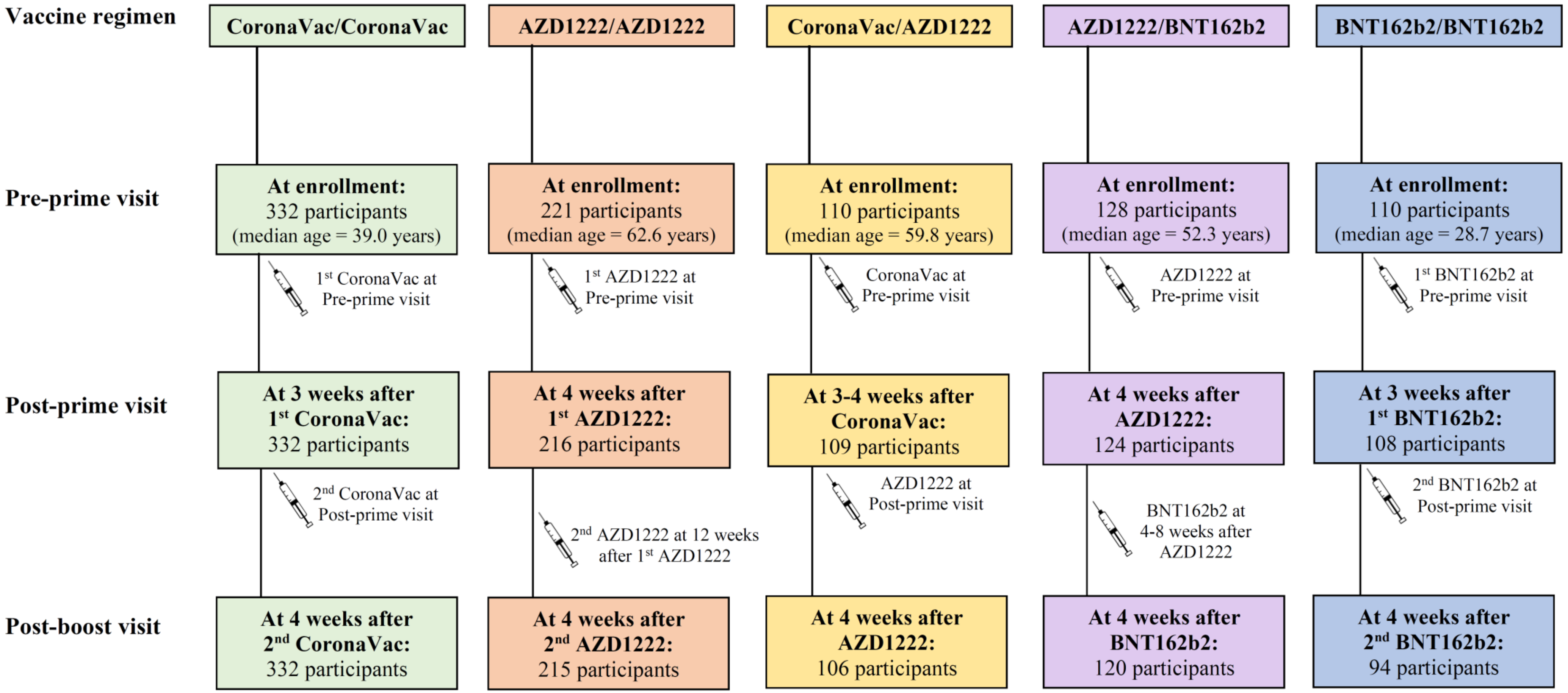

Supplementary Figure S1. Diagram of study participants.

Abbreviations: AZD1222 vaccine, the Oxford-AstraZeneca COVID-19 vaccine; BNT162b2, Pfizer-BioNTech vaccine.

(A). Adults aged < 60 years

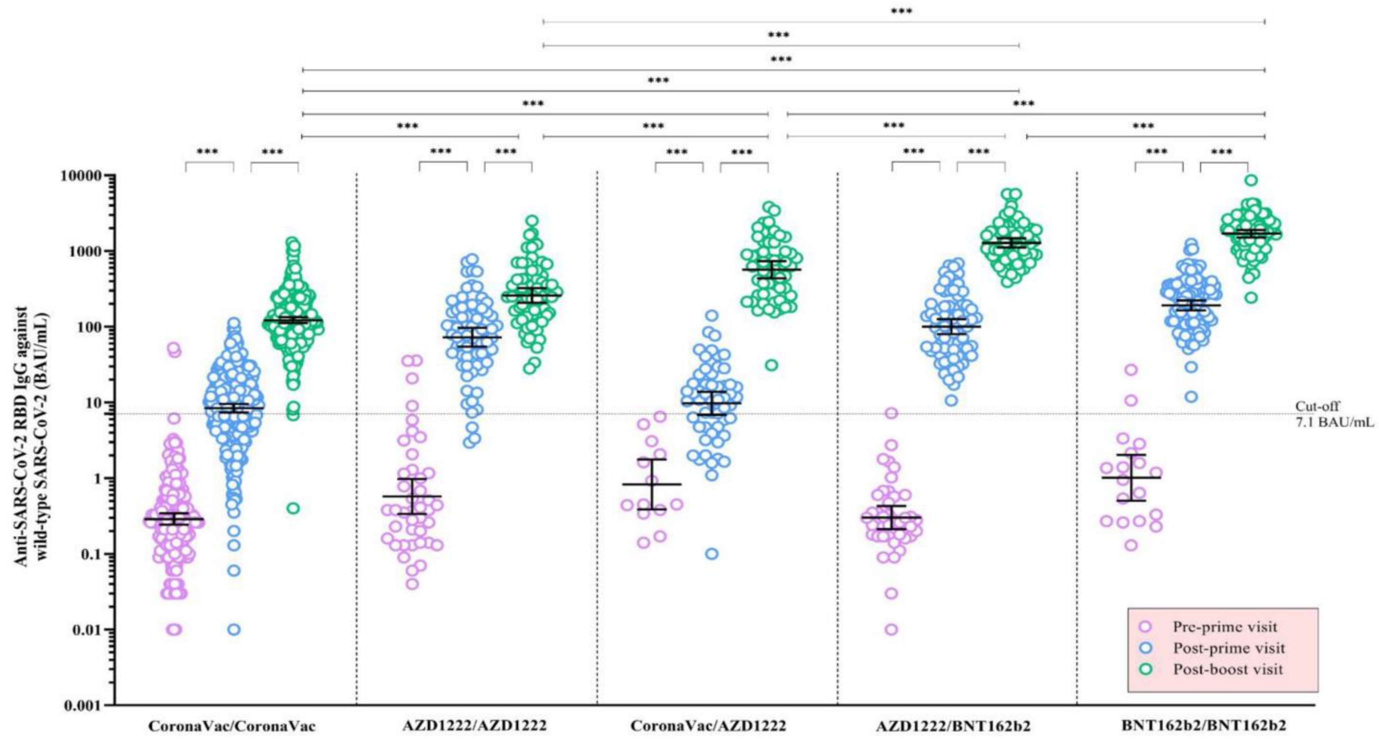

	CoronaVac/CoronaVac			AZD1222/AZD1222			CoronaVac/AZD1222			AZD1222/BNT162b2			BNT162b2/BNT162b2		
Number of participants	332	332	332	73	72	72	55	54	53	73	73	73	110	108	94
GMC (BAU/mL) (95%CI)	0.3 (0.2-0.3)	8.4 (7.4-9.5)	122.2 (111.4-134.0)	0.5 (0.3-1.0)	72.6 (54.6-96.5)	258.3 (207.2-321.8)	0.8 (0.4-1.8)	9.7 (6.9-13.7)	566.4 (435.0-737.6)	0.3 (0.2-0.4)	100.3 (79.9-125.9)	1279.1 (1111.8-1471.5)	1.0 (0.5-2.0)	191.2 (164.3-222.4)	1697.6 (1510.9-1907.4)
Number of seropositive participants (%)	2 (0.6)	199 (59.9)	330 (99.4)	4 (5.5)	69 (95.8)	72 (100.0)	0 (0.0)	36 (66.7)	53 (100.0)	1 (1.4)	73 (100.0)	73 (100.0)	2 (1.8)	108 (100.0)	94 (100.0)

(B). Elderly aged ≥ 60 years

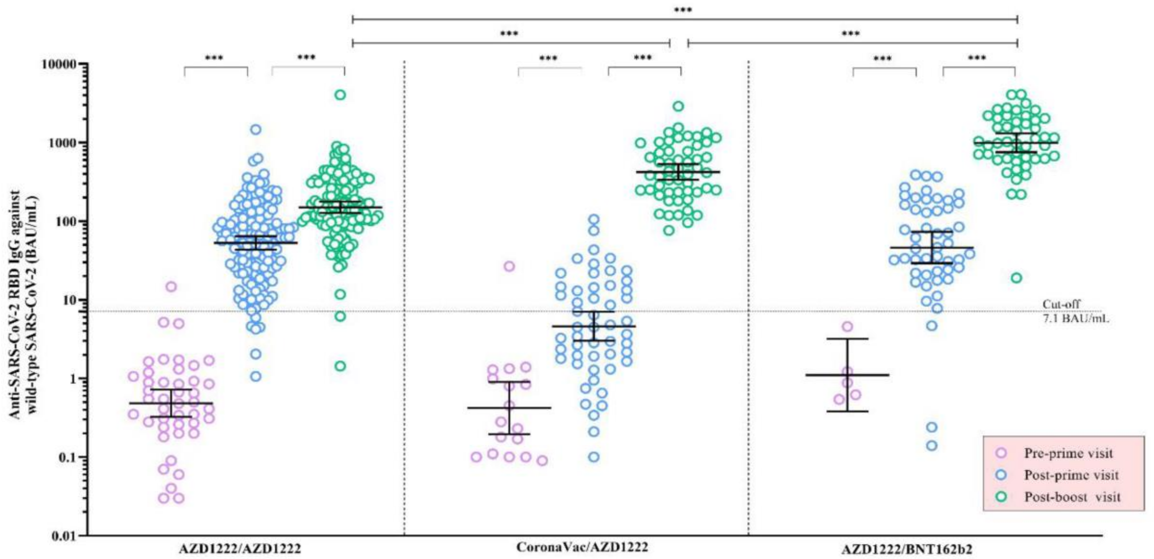

	AZD1222/AZD1222			CoronaVac/AZD1222			AZD1222/BNT162b2		
Number of participants	148	144	143	55	55	53	55	51	47
GMC (BAU/mL) (95%CI)	0.5 (0.3-0.7)	53.0 (43.5-64.5)	150.0 (127.4-176.6)	0.4 (0.2-0.9)	4.6 (3.0-7.1)	421.4 (335.0-530.1)	1.1 (0.4-3.2)	46.6 (29.3-73.4)	989.3 (752.6-1300.5)
Number of seropositive participants (%)	1 (0.7)	138 (95.8)	141 (98.6)	1 (1.8)	22 (40.0)	53 (100.0)	0 (0.0)	46 (90.2)	47 (100.0)

Supplementary Figure S2. Anti-SARS-CoV-2 receptor-binding domain immunoglobulin G against wild-type SARS-CoV-2 following a primary series of COVID-19 vaccination, stratified by the COVID-19 vaccine regimen and participant's age group. (A) For adults aged < 60 years; (B) For elderly aged ≥ 60 years. Data points are the reciprocals of individual participants. The geometric mean concentrations (GMC) and the corresponding 95% confidence intervals (95% CI) of anti-SARS-CoV-2 receptor-binding domain immunoglobulin G against wild-type SARS-CoV-2 among all participants at the pre-prime, post-prime, and post-boost visits are shown as black solid lines. Black dotted lines indicate the cut-off values. The mixed-effects log-linear regression analysis was performed to compare the GMCs of anti-SARS-CoV-2 receptor-binding domain immunoglobulin G between visits within each COVID-19 vaccine regimen. The log-linear regression analysis was performed to compare the GMCs of anti-SARS-CoV-2 receptor-binding domain immunoglobulin G between COVID-19 vaccine regimens. *** indicates $P < 0.001$ for the comparison of the GMCs of anti-SARS-CoV-2 receptor-binding domain immunoglobulin G between indicated visits within each vaccine regimen, and between indicated vaccine regimens.

Abbreviations: AZD1222 vaccine, the Oxford-AstraZeneca COVID-19 vaccine; BAU, binding antibody unit; BNT162b2, Pfizer-BioNTech vaccine; COVID-19, coronavirus disease 2019; GMC, geometric mean concentration; IgG, immunoglobulin G; RBD, receptor-binding domain; SARS-CoV-2, severe acute respiratory syndrome coronavirus-2; 95% CI, 95% confidence interval.

(A). Adults aged < 60 years

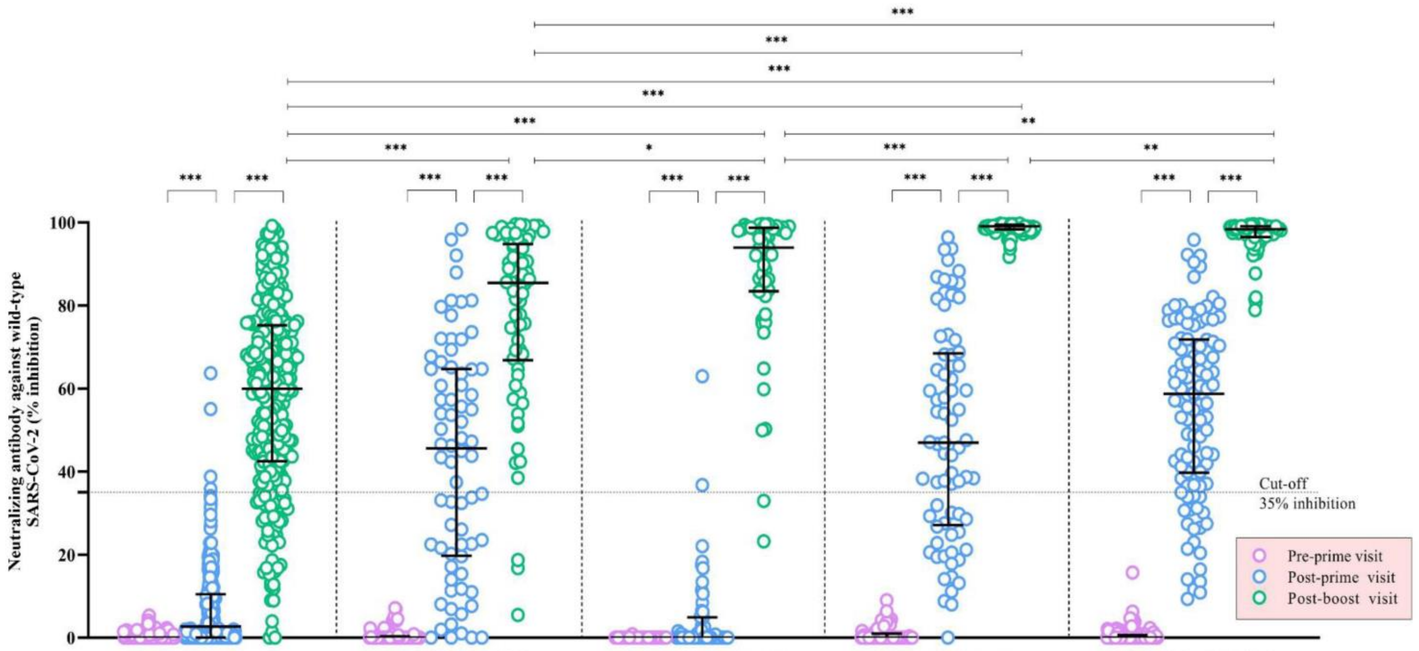

	CoronaVac/CoronaVac			AZD1222/AZD1222			CoronaVac/AZD1222			AZD1222/BNT162b2			BNT162b2/BNT162b2		
Number of participants	332	332	332	73	72	72	55	54	53	73	73	73	110	108	94
Median (% inhibition) (IQR)	0.0	2.7 (0.0-10.4)	59.9 (42.5-75.2)	0.0	45.6 (19.9-64.7)	85.4 (67.0-94.7)	0.0	0.0 (0.0-4.9)	94.0 (83.5-98.6)	0.0	47.0 (27.5-68.2)	99.1 (98.4-99.4)	0.0	58.8 (39.8-71.8)	98.4 (96.5-99.1)
Number of seropositive participants (%)	0 (0.0)	4 (1.2)	275 (82.8)	0 (0.0)	41 (56.9)	69 (95.8)	0 (0.0)	2 (3.7)	51 (96.2)	0 (0.0)	49 (67.1)	73 (100.0)	0 (0.0)	87 (80.6)	94 (100.0)

(B). Elderly aged ≥ 60 years

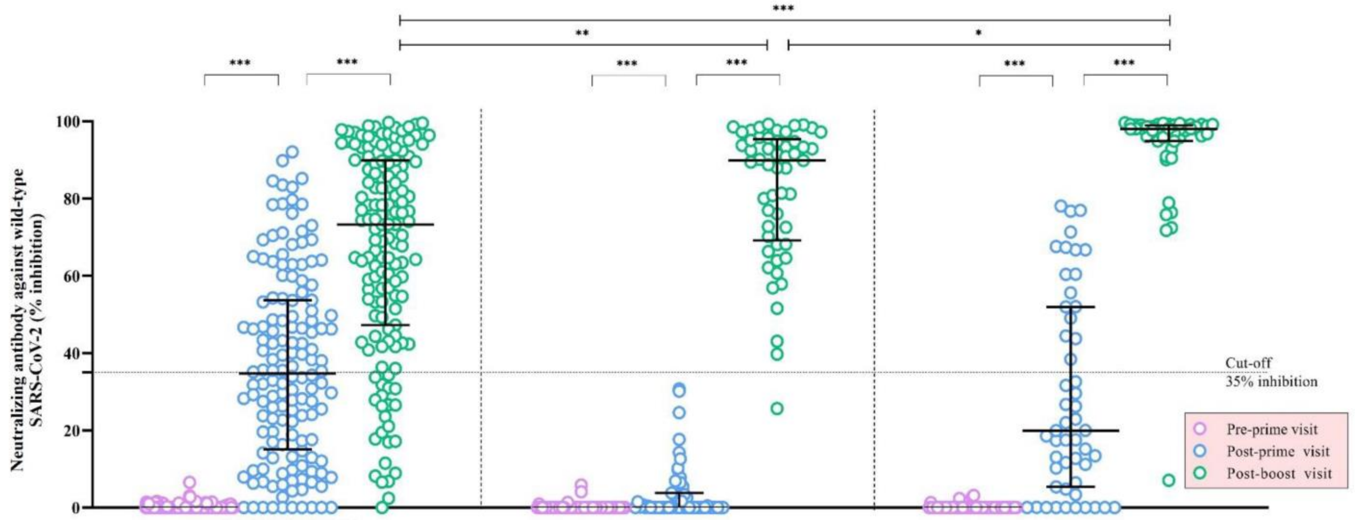

	AZD1222/AZD1222			CoronaVac/AZD1222			AZD1222/BNT162b2		
Number of participants	148	144	143	55	55	53	55	51	47
Median (% inhibition) (IQR)	0.0	34.7 (15.5-53.7)	73.3 (47.2-89.9)	0.0	0.0 (0.0-3.8)	89.9 (70.2-95.0)	0.0	19.9 (5.4-52.0)	98.0 (94.9-99.0)
Number of seropositive participants (%)	0 (0.0)	72 (50.0)	120 (83.9)	0 (0.0)	0 (0.0)	52 (98.1)	0 (0.0)	17 (33.3)	46 (97.9)

Supplementary Figure S3. Neutralizing antibody against wild-type SARS-CoV-2 following a primary series of COVID-19 vaccination, stratified by the COVID-19 vaccine regimen and participant's age group. (A) For adults aged < 60 years; (B) For elderly aged ≥ 60 years. Data points are the reciprocals of individual participants. The median values of neutralizing antibody (NAb) and the corresponding interquartile ranges (IQR) against wild-type SARS-CoV-2 among all participants at the pre-prime, post-prime, and post-boost visits are shown as black solid lines. Black dotted lines indicate the cut-off values. The Wilcoxon signed-rank test was performed to compare the medians of NAb against wild-type SARS-CoV-2 between visits within each COVID-19 vaccine regimen. The median regression analysis was performed to compare the medians of NAb against wild-type SARS-CoV-2 between COVID-19 vaccine regimens. * indicates $P < 0.05$; ** indicates $P < 0.01$, and *** indicates $P < 0.001$ for the comparison of NAb against wild-type SARS-CoV-2 between visits within each vaccine regimen, and between indicated vaccine regimens.

Abbreviations: AZD1222 vaccine, the Oxford-AstraZeneca COVID-19 vaccine; BNT162b2, Pfizer-BioNTech vaccine; COVID-19, coronavirus disease 2019; IQR, interquartile range; NAb, neutralizing antibody; SARS-CoV-2, severe acute respiratory syndrome coronavirus-2.

(A). Adults aged < 60 years

**Number of participants**	92	92	92	92	59	59	59	59	59	53	53	53	53	53	60	60	60	60	60	94	94	94	94	94
**Median (% inhibition) (IQR)**	74 (64-85)	36 (27-46)	39 (30-47)	66 (56-76)	91 (64-98)	69 (28-87)	49 (21-71)	80 (39-93)	21 (0-30)	94 (69-98)	67 (34-90)	47 (27-71)	84 (56-95)	10 (0-26)	99 (98-99)	97 (93-98)	92 (82-96)	98 (96-98)	52 (25-62)	97 (97-98)	90 (85-95)	83 (77-89)	96 (93-97)	10 (3-16)
**Number of seropositive participants (%)**	91 (98.9)	62 (67.4)	71 (77.2)	89 (96.7)	57 (96.6)	43 (72.9)	39 (66.1)	47 (79.7)	16 (27.1)	50 (94.3)	40 (75.5)	39 (73.6)	48 (90.6)	10 (18.9)	58 (96.7)	58 (96.7)	58 (96.7)	58 (96.7)	43 (71.7)	94 (100.0)	94 (100.0)	94 (100.0)	94 (100.0)	8 (8.5)

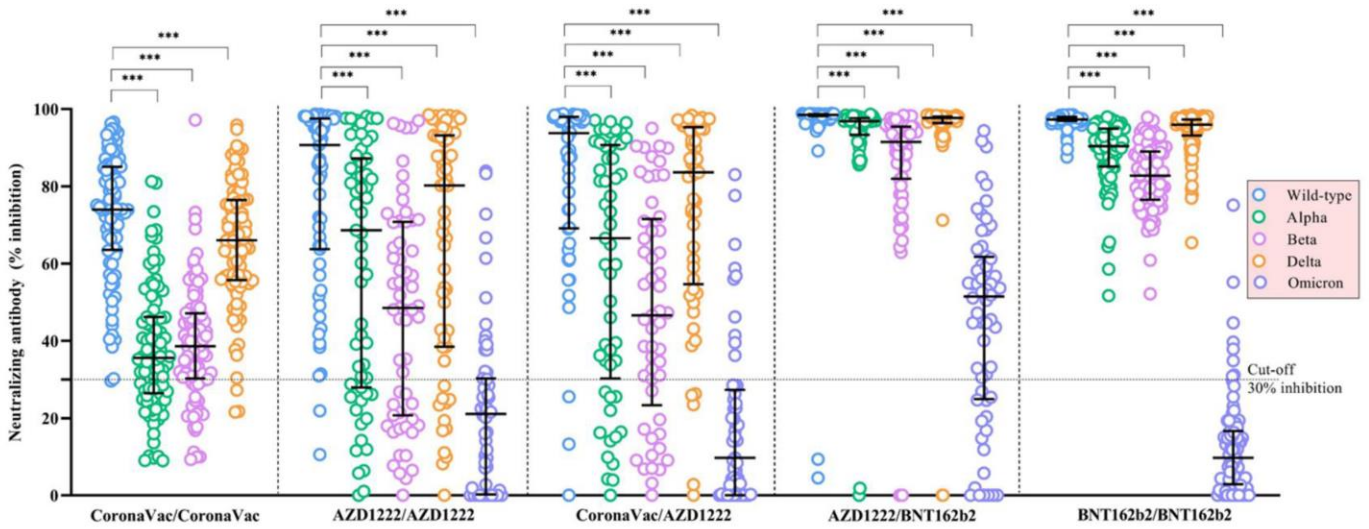

	WT	A	B	D		WT	A	B	D	O		WT	A	B	D	O		WT	A	B	D	O		WT	A	B	D	O
**WT**	Ref 1					***						***						***						***				
**A**		Ref 1					***						***						***						***			
**B**			Ref 1					ns						ns						***						***		
**D**				Ref 1					*						**						***						***	
**O**										Ref 2						ns						***						**

(B). Elderly Aged ≥ 60 years

**Number of participants**	61	61	61	61	61	53	53	53	53	53	47	47	47	47	47
**Median (% inhibition) (IQR)**	75 (45-93)	42 (15-67)	18 (8-44)	45 (28-84)	21 (14-30)	80 (67-94)	39 (27-70)	34 (20-54)	60 (44-86)	13 (1-23)	98 (96-98)	90 (69-95)	79 (58-91)	94 (77-98)	2 (0-8)
**Number of seropositive participants (%)**	50 (82.0)	39 (63.9)	19 (31.2)	43 (70.5)	16 (26.2)	52 (98.1)	33 (62.3)	30 (56.6)	48 (90.6)	9 (17.0)	46 (97.9)	45 (95.7)	46 (97.9)	46 (97.9)	2 (4.3)

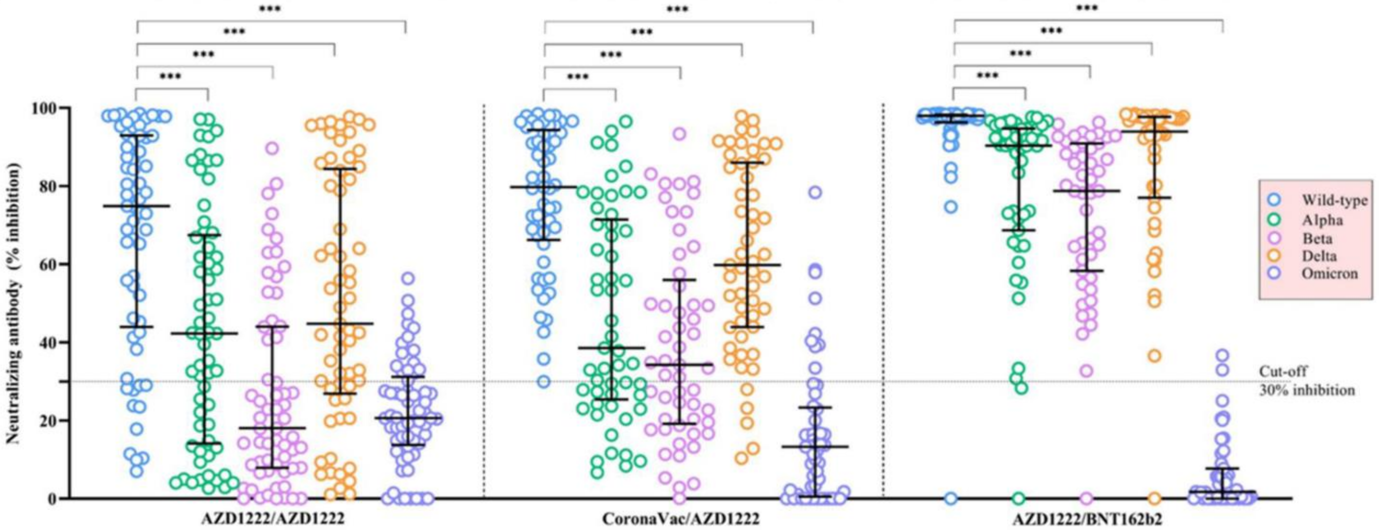

	WT	A	B	D	O		WT	A	B	D	O		WT	A	B	D	O
**WT**	Ref 2						ns						***				
**A**		Ref 2						ns						***			
**B**			Ref 2						**						***		
**D**				Ref 2						ns						***	
**O**					Ref 2						*						***

Supplementary Figure S4. Neutralizing antibody, based on the in-house surrogate virus neutralization test, against wild-type SARS-CoV-2 and other circulating variants of concern following the completion of a primary series of COVID-19 vaccination, stratified by the COVID-19 vaccine regimen and participant's age group. (A) For adults aged < 60 years; (B) For elderly aged ≥ 60 years. Data points are the reciprocals of individual participants. The median values of neutralizing antibody (NAb; an in-house surrogate virus neutralization test) and the corresponding interquartile ranges (IQR) against wild-type SARS-CoV-2 and other circulating variants of concern following the completion of a primary series of COVID-19 vaccination are shown as black solid lines. Black dotted lines indicate the cut-off values. The Wilcoxon signed-rank test was performed to compare the medians of NAb between wild-type SARS-CoV-2 and the indicated variant of concern within each COVID-19 vaccine regimen, and the results are shown in the area above the figure. The median regression analysis was performed to compare the medians of NAb to wild-type SARS-CoV-2 or the indicated variant of concern between COVID-19 vaccine regimens, and the results are shown in the table below the figure. For adults aged < 60 years (Supplementary Figure S4A), the comparisons NAb-WT, NAb-Alpha, NAb-Beta, and NAb-Delta were conducted between the indicated COVID-19 vaccine regimen and the homologous CoronaVac regimen (the reference vaccine regimen, Ref 1), whereas the comparisons of NAb-Omicron were conducted between the

indicated vaccine regimen and the homologous AZD1222 regimen (the reference vaccine regimen, Ref 2). For elderly aged ≥ 60 years (Supplementary Figure S4B), the comparisons of NAb-WT, NAb-Alpha, NAb-Beta, NAb-Delta, and NAb-Omicron were conducted between the indicated vaccine regimen and the homologous AZD1222 regimen (the reference vaccine regimen, Ref 2). * indicates $P < 0.05$; ** indicates $P < 0.01$; *** indicates $P < 0.001$; and ns indicates non-significant for the comparison of NAb between wild-type SARS-CoV-2 and the indicated variant of concern within each COVID-19 vaccine regimen, and the comparison of NAb against indicated variant of concern between vaccine regimens.

Abbreviations: A, the SARS-CoV-2 Alpha variant; AZD1222 vaccine, the Oxford-AstraZeneca COVID-19 vaccine; B, the SARS-CoV-2 Beta variant; BNT162b2, Pfizer-BioNTech vaccine; COVID-19, coronavirus disease 2019; D, the SARS-CoV-2 Delta variant; IQR, interquartile range; NAb, neutralizing antibody; NAb-Alpha, neutralizing antibody against Alpha variant; NAb-Beta, neutralizing antibody against Beta variant; NAb-Delta, neutralizing antibody against Delta variant; NAb-O, neutralizing antibody against Omicron variant; NAb-WT, neutralizing antibody against wild-type SARS-CoV-2; O, the SARS-CoV-2 Omicron variant; Ref 1, the homologous CoronaVac regimen as the reference COVID-19 vaccine regimen for the comparison; Ref 2, the homologous AZD1222 regimen as the reference COVID-19 vaccine regimen for the comparison; SARS-CoV-2, severe acute respiratory syndrome coronavirus-2.
