# Supplementary material for: Comparison of Immunogenicity and Reactogenicity of Five Primary Series of COVID-19 Vaccine Regimens against Circulating SARS-CoV-2 Variants of Concern among Healthy Thai Populations
Source: Vaccines (Basel). 2023 Mar 1;11(3):564. doi: 10.3390/vaccines11030564 (PMC10051392; doi:10.3390/vaccines11030564)
Supplement: Supplementary file 1 [file vaccines-11-00564-s001.zip › Supplementary_Tables.pdf]

Supplementary Table S1. The proportion of seropositive participants based on anti-SARS-CoV-2 receptor-binding domain immunoglobulin G against wild-type SARS-CoV-2 following a primary series of COVID-19 vaccination, stratified by the COVID-19 vaccine regimen.

**Study participant and study visit**	**CoronaVac/CoronaVac**			**AZD1222/AZD1222**			**CoronaVac/AZD1222**			**AZD1222/BNT162b2**			**BNT162b2/BNT162b2**		
	**Seropositive participants** (n/N, %)	**Proportion difference[a]** (%, 95% CI)	***P*[a]**	**Seropositive participants** (n/N, %)	**Proportion difference[a]** (%, 95% CI)	***P*[a]**	**Seropositive participants** (n/N, %)	**Proportion difference[a]** (%, 95% CI)	***P*[a]**	**Seropositive participants** (n/N, %)	**Proportion difference[a]** (%, 95% CI)	***P*[a]**	**Seropositive participants** (n/N, %)	**Proportion difference[a]** (%, 95% CI)	***P*[a]**
All participants															
Pre-prime visit	2/332 (0.6)			5/221 (2.3)			1/110 (0.9)			1/128 (0.8)			2/110 (1.8)		
Post-prime visit	199/332 (59.9)	59.3 (53.8-64.9)	<0.001	207/216 (95.8)	93.5 (89.8-97.3)	<0.001	58/109 (53.2)	52.3 (42.0-63.6)	<0.001	119/124 (96.0)	95.2 (90.6- 99.7)	<0.001	108/108 (100)	98.2 (94.7-101.6)	<0.001
Post-boost visit	330/332 (99.4)	39.5 (33.9-45.0)	<0.001	213/215 (99.1)	3.3 (0.1-6.5)	0.02	106/106 (100)	46.8 (36.7-57.6)	<0.001	120/120 (100)	4.0 (-0.2 to 8.6)	0.03	94/94 (100)	0.0	NA
Adults aged < 60 years old															
Pre-prime visit	2/332 (0.6)			4/73 (5.5)			0/55 (0)			1/73 (1.4)			2/110 (1.8)		
Post-prime visit	199/332 (59.9)	59.3 (53.8-64.9)	<0.001	69/72 (95.8)	90.3 (82.0-98.5)	<0.001	36/54 (66.7)	66.7 (52.2- 81.1)	<0.001	73/73 (100)	98.6 (94.6-102.7)	<0.001	108/108 (100)	98.2 (94.7-101.6)	<0.001
Post-boost visit	330/332 (99.4)	39.5 (33.9-45.0)	<0.001	72/72 (100)	4.2 (-1.9 to 10.3)	0.08	53/53 (100)	33.3 (19.3-48.6)	<0.001	73/73 (100)	0.0	NA	94/94 (100)	0.0	NA
Elderly aged ≥ 60 years old															
Pre-prime visit	-	-	-	1/148 (0.7)			1/55 (1.8)			0/55 (0.0)			-	-	-
Post-prime visit	-	-	-	138/144 (95.8)	95.1 (90.9-99.3)	<0.001	22/55 (40.0)	38.2 (23.5-52.8)	<0.001	46/51 (90.2)	90.2 (80.1-100.3)	<0.001	-	-	-
Post-boost visit	-	-	-	141/143 (98.6)	2.8 (-1.2 to 6.9)	0.10	53/53 (100)	60.0 (45.3-75.4)	<0.001	47/47 (100)	10.8 (-0.3 to 22.0)	0.03	-	-	-

Abbreviations: AZD1222 vaccine, the Oxford-AstraZeneca COVID-19 vaccine; BNT162b2, Pfizer-BioNTech vaccine; COVID-19, coronavirus disease 2019; NA, not applicable; SARS-CoV-2, severe acute respiratory syndrome coronavirus-2; 95% CI, 95% confidence interval.

[a]The McNemar's test was performed to compare the proportions of seropositive participants, based on anti-SARS-CoV-2 receptor-binding domain immunoglobulin G, between the indicated visit and the previous visit within each COVID-19 vaccine regimen.

Supplementary Table S2. The proportion of participants with positive neutralizing activity against wild-type SARS-CoV-2 following a primary series of COVID-19 vaccination, stratified by the COVID-19 vaccine regimen.

Study participant and study visit	CoronaVac/CoronaVac			AZD1222/AZD1222			CoronaVac/AZD1222			AZD1222/BNT162b2			BNT162b2/BNT162b2		
	**Seropositive participants** (n/N, %)	**Proportion difference[a]** (%, 95% CI)	***P*[a]**	**Seropositive participants** (n/N, %)	**Proportion difference[a]** (%, 95% CI)	***P*[a]**	**Seropositive participants** (n/N, %)	**Proportion difference[a]** (%, 95% CI)	***P*[a]**	**Seropositive participants** (n/N, %)	**Proportion difference[a]** (%, 95% CI)	***P*[a]**	**Seropositive participants** (n/N, %)	**Proportion difference[a]** (%, 95% CI)	***P*[a]**
All participants															
Pre-prime visit	0/332 (0)			0/221 (0)			0/110 (0)			0/128 (0)			0/110 (0)		
Post-prime visit	4/332 (1.2)	1.2 (-0.3 to 2.7)	0.05	113/216 (52.3)	52.3 (45.2-59.4)	<0.001	2/109 (1.8)	1.8 (-1.6 to 5.3)	0.16	66/124 (53.2)	53.2 (43.6-62.8)	<0.001	87/108 (80.6)	80.6 (72.2-88.9)	<0.001
Post-boost visit	275/332 (82.8)	81.6 (77.2-86.1)	<0.001	189/215 (87.9)	35.6 (28.8-43.0)	<0.001	103/106 (97.2)*	95.4 (90.3-100.2)	<0.001	119/120 (99.2)*	46.0 (34.8-54.3)	<0.001	94/94 (100)*	19.4 (11.9-30.6)	<0.001
Adults aged < 60 years old															
Pre-prime visit	0/332 (0)			0/73 (0)			0/55 (0)			0/73 (0)			0/110 (0)		
Post-prime visit	4/332 (1.2)	1.2 (-0.3 to 2.7)	0.05	41/72 (56.9)	56.9 (444.1-69.8)	<0.001	2/54 (3.7)	3.7 (-3.2 to 10.6)	0.16	49/73 (67.1)	67.1 (55.0-79.3)		87/108 (80.6)	80.6 (72.2-88.9)	<0.001
Post-boost visit	275/332 (82.8)	81.6 (77.2-86.1)	<0.001	69/72 (95.8)*	38.9 (24.7-51.4)	<0.001	51/53 (96.2)*	92.5 (83.5-1.01)	<0.001	73/73 (100)*	32.9 (20.7-45.0)		94/94 (100)*	19.4 (11.9-30.6)	<0.001
Elderly aged ≥ 60 years old															
Pre-prime visit	-	-	-	0/148 (0)			0/55 (0)			0/55 (0)			-	-	-
Post-prime visit	-	-	-	72/144 (50.0)	50.0 (41.1-58.9)	<0.001	0/55 (0)	0.0	NA	17/51 (33.3)	33.3 (18.4-48.2)		-	-	-
Post-boost visit	-	-	-	120/143 (83.9)	33.9 (24.4-43.7)	<0.001	52/53 (98.1)[†]	98.1 (92.6-103.7)	<0.001	46/47 (97.9)[†]	64.6 (46.9-79.2)	<0.001	-	-	-

Abbreviations: AZD1222 vaccine, the Oxford-AstraZeneca COVID-19 vaccine; BNT162b2, Pfizer-BioNTech vaccine; COVID-19, coronavirus disease 2019; SARS-CoV-2, severe acute respiratory syndrome coronavirus-2; 95% CI, 95% confidence interval.

[a]The McNemar's test was performed to compare the proportions of participants with positive neutralizing activity against wild-type SARS-CoV-2 between the indicated visit and the previous visit within each COVID-19 vaccine regimen.

*Indicates a significant difference ($P < 0.01$) of the proportion of participants with detectable neutralizing activity against wild-type SARS-CoV-2 after the completion of a primary series of an indicated COVID-19 vaccination compared with the homologous CoronaVac vaccine regimen, evaluated by the Chi-squared test.

[†]Indicates a significant difference ($P < 0.01$) of the proportion of participants with detectable neutralizing activity against wild-type SARS-CoV-2 after the completion of a primary series of an indicated COVID-19 vaccination compared with the homologous AZD1222 vaccine regimen, evaluated by the Chi-squared test.

Supplementary Table S3. The proportion of participants with positive neutralizing activity, based on the in-house surrogate virus neutralization test, against wild-type and other circulating variants of concern of SARS-CoV-2 following the completion of a primary series of COVID-19 vaccination, stratified by the COVID-19 vaccine regimen.

Study participant and SARS-CoV-2 variant	CoronaVac/CoronaVac			AZD1222/AZD1222			CoronaVac/AZD1222			AZD1222/BNT162b2			BNT162b2/BNT162b2		
	Seropositive participants (n/N, %)	Proportion difference[a] (%)	*P*[a]	Seropositive participants (n/N, %)	Proportion difference[a] (%)	*P*[a]	Seropositive participants (n/N, %)	Proportion difference[a] (%)	*P*[a]	Seropositive participants (n/N, %)	Proportion difference[a] (%)	*P*[a]	Seropositive participants (n/N, %)	Proportion difference[a] (%)	*P*[a]
All participants															
Wild-type	91/92 (98.9)	Ref	Ref	107/120 (89.2)*	Ref	Ref	102/106 (96.2)	Ref	Ref	104/107 (97.2)	Ref	Ref	94/94 (100)	Ref	Ref
Alpha	62/92 (67.4)	-31.5 (-42.1 to -20.9)	<0.001	82/120 (68.3)	-20.9 (-28.9 to -12.7)	<0.001	73/106 (68.9)	-27.3 (-36.8 to -17.9)	<0.001	103/107 (96.3)*	-0.9 (-3.7 to 1.8)	0.32	94/94 (100)*	0	NA
Beta	71/92 (77.2)	-21.7 (-31.3 to -12.2)	<0.001	58/120 (48.3)*	-40.9 (-50.5 to -31.2)	<0.001	69/106 (65.1)	-31.1 (-40.9 to -21.4)	<0.001	104/107 (97.2)*	0	NA	94/94 (100)*	0	NA
Delta	89/92 (96.7)	-2.2 (-6.2 to 1.9)	0.16	90/120 (75.0)*	-14.2 (-21.2 to -7.1)	<0.001	96/106 (90.6)	-5.6 (-11.0 to -0.3)	0.01	104/107 (97.2)	0	NA	94/94 (100)	0	NA
Omicron	-	-	-	32/120 (26.7)	-62.5 (-72.0 to -53.0)	<0.001	19/106 (17.9)	-78.3 (-87.1 to -69.5)	<0.001	45/107 (42.1)†	-55.1 (-65.5 to -44.8)	<0.001	8/94 (8.5)†	-91.5 (-98.2 to -84.8)	<0.001
Adults aged < 60 years old															
Wild-type	91/92 (98.9)	Ref	Ref	57/59 (96.6)	Ref	Ref	50/53 (94.3)	Ref	Ref	58/60 (96.7)	Ref	Ref	94/94 (100)	Ref	Ref
Alpha	62/92 (67.4)	-31.5 (-42.1 to -20.9)	<0.001	43/59 (72.9)	-23.7 (-36.3 to -11.2)	<0.001	40/53 (75.5)	-18.8 (-31.3 to -6.4)	0.002	58/60 (96.7)*	0	NA	94/94 (100)*	0	NA
Beta	71/92 (77.2)	-21.7 (-31.3 to -12.2)	<0.001	39/59 (66.1)	-30.5 (-44.0 to -17.1)	<0.001	39/53 (73.6)	-20.7 (-33.6 to -7.9)	0.001	58/60 (96.7)*	0	NA	94/94 (100)*	0	NA
Delta	89/92 (96.7)	-2.2 (-6.2 to 1.9)	0.16	47/59 (79.7)*	-16.9 (-28.2 to -5.7)	0.002	48/53 (90.6)	-3.7 (-10.8 to 3.2)	0.16	58/60 (96.7)	0	NA	94/94 (100)	0	NA
Omicron		-	-	16/59 (27.1)	-69.5 (-82.9 to -56.0)	<0.001	10/53 (18.9)	-75.4 (-88.9 to -62.0)	<0.001	43/60 (71.7)†	-25.0 (-37.6 to -12.4)	<0.001	8/94 (8.5)†	-91.5 (-98.2 to -84.8)	<0.001
Elderly aged ≥ 60 years old															
Wild-type	-	-	-	50/61 (82.0)	Ref	Ref	52/53 (98.1)†	Ref	Ref	46/47 (97.9)†	Ref	Ref	-	-	-
Alpha	-	-	-	39/61 (63.9)	-18.1 (-29.3 to -6.7)	0.001	33/53 (62.3)	-35.8 (-50.6 to -21.1)	<0.001	45/47 (95.7)†	-2.2 (-8.4 to 4.1)	0.32	-	-	-
Beta	-	-	-	19/61 (31.1)	-50.9 (-65.0 to -36.6)	<0.001	30/53 (56.6)†	-41.5 (-56.7 to -26.4)	<0.001	46/47 (97.9)†	0	NA	-	-	-
Delta	-	-	-	43/61 (70.5)	-11.5 (-21.1 to -1.8)	0.008	48/53 (90.6)†	-7.5 (-16.5 to 1.5)	0.04	46/47 (97.9)†	0	NA	-	-	-
Omicron	-	-	-	16/61 (26.2)	-55.8 (-69.8 to -41.6)	<0.001	9/53 (17.0)	-81.1 (-93.6 to -68.7)	<0.001	2/47 (4.3)†	-93.6 (-102.7 to -84.5)	<0.001	-	-	-

Abbreviations: AZD1222 vaccine, the Oxford-AstraZeneca COVID-19 vaccine; BNT162b2, Pfizer-BioNTech vaccine; COVID-19, coronavirus disease 2019; NA, not applicable; SARS-CoV-2, severe acute respiratory syndrome coronavirus-2.

[a]The McNemar's test was performed to compare the proportions of participants with positive neutralizing activity, based on the in-house surrogate virus neutralization test, between the wild-type SARS-CoV-2 and the indicated variant of concern within each COVID-19 vaccine regimen.

*Indicates a significant difference ($P < 0.01$) of the proportion of participants with positive neutralizing activity, based on the in-house surrogate virus neutralization test, against wild-type and other circulating variants of concern of SARS-CoV-2 after the completion of a primary series of an indicated COVID-19 vaccination compared with the homologous CoronaVac vaccine regimen, evaluated by the Chi-squared test.

[†]Indicates a significant difference ($P < 0.01$) of the proportion of participants with positive neutralizing activity, based on the in-house surrogate virus neutralization test, against wild-type and other circulating variants of concern of SARS-CoV-2 after the completion of a primary series of an indicated COVID-19 vaccination compared with the homologous AZD1222 vaccine regimen, evaluated by the Chi-squared test.
